# Epistemic Communities under Active Inference

**DOI:** 10.3390/e24040476

**Published:** 2022-03-29

**Authors:** Mahault Albarracin, Daphne Demekas, Maxwell J. D. Ramstead, Conor Heins

**Affiliations:** 1 Department of Cognitive Computing, Université du Québec a Montreal, Montreal, QC H2K 4M1, Canada; albarracin.mahault@courrier.uqam.ca; 2 VERSES Labs, Los Angeles, CA 90016, USA; maxwell.d.ramstead@gmail.com; 3 Department of Computing, Imperial College London, London SW7 5NH, UK; daphnedemekas@gmail.com; 4 Wellcome Centre for Human Neuroimaging, University College London, London WC1N 3AR, UK; 5 Department of Collective Behaviour, Max Planck Institute of Animal Behaviour, 78315 Radolfzell am Bodensee, Germany; 6 Centre for the Advanced Study of Collective Behaviour, University of Konstanz, 78464 Konstanz, Germany; 7 Department of Biology, University of Konstanz, 78457 Konstanz, Germany

**Keywords:** epistemic community, social media, active inference, opinion dynamics

## Abstract

The spread of ideas is a fundamental concern of today’s news ecology. Understanding the dynamics of the spread of information and its co-option by interested parties is of critical importance. Research on this topic has shown that individuals tend to cluster in echo-chambers and are driven by confirmation bias. In this paper, we leverage the active inference framework to provide an in silico model of confirmation bias and its effect on echo-chamber formation. We build a model based on active inference, where agents tend to sample information in order to justify their own view of reality, which eventually leads to them to have a high degree of certainty about their own beliefs. We show that, once agents have reached a certain level of certainty about their beliefs, it becomes very difficult to get them to change their views. This system of self-confirming beliefs is upheld and reinforced by the evolving relationship between an agent’s beliefs and observations, which over time will continue to provide evidence for their ingrained ideas about the world. The epistemic communities that are consolidated by these shared beliefs, in turn, tend to produce perceptions of reality that reinforce those shared beliefs. We provide an active inference account of this community formation mechanism. We postulate that agents are driven by the epistemic value that they obtain from sampling or observing the behaviours of other agents. Inspired by digital social networks like Twitter, we build a generative model in which agents generate observable social claims or posts (e.g., ‘tweets’) while reading the socially observable claims of other agents that lend support to one of two mutually exclusive abstract topics. Agents can choose which other agent they pay attention to at each timestep, and crucially who they attend to and what they choose to read influences their beliefs about the world. Agents also assess their local network’s perspective, influencing which kinds of posts they expect to see other agents making. The model was built and simulated using the freely available Python package pymdp. The proposed active inference model can reproduce the formation of echo-chambers over social networks, and gives us insight into the cognitive processes that lead to this phenomenon.

## 1. Introduction

### 1.1. Confirmation Bias and Conformity

The practice of exchanging ideas, sharing concepts and values between different minds, is a fundamental process that allows humans and other living agents to coordinate and operate socially. By sharing ideas, individuals and communities can better pursue their pragmatic goals and improve their understanding of the world and each other. Humans are compulsory cooperators [1]: human survival itself is predicated on the ability to access and leverage bodies of accumulated cultural knowledge. Over the course of evolutionary history, humans have developed an exquisitely sensitive capacity to discriminate reliable sources of information from unreliable ones, and to learn from other relevant human agents to improve their understanding or model of their world [2,3].

This epistemic process is not, however, without its flaws. There is evidence that humans process information by reasoning heuristically, which is hypothesised to limit the consumption of energy and facilitate rapid decision-making [4,5]. One such heuristic is confirmation bias, which implies that, all other things being equal, individuals prefer sticking to their own beliefs over changing their minds [6]. There is an extensive literature documenting the phenomenon of *confirmation bias* and its relation to cognitive dissonance. Individuals faced with information that conflicts with their core beliefs may be prone to cognitive dissonance, which is experienced as undesirable [7,8,9]. Tolerance for cognitive dissonance varies across individuals, but in general, the phenomenon significantly influences decision-making [8,10]. To avoid such dissonance, individuals tend to selectively seek information from ‘others like me’, others who they expect will share similar ideas, concepts, and values [8]. Confirmation bias has a social influence; in particular, individuals prefer sampling data from their in-group, and will seek to confirm their own ideas by foraging for confirmatory information from their in-group [11,12]. To make sure that they have access to other like-minded allies, agents are more likely to choose to belong to communities where their deeply held beliefs are promoted and shared, which limits the cognitive effort that is already expanded in the foraging of information [10]. In-group delivery of information influences how strongly this information is integrated, especially if group membership is important for the individual [13]. This sampling extends beyond other agents, to choice of media and environment. For instance, individuals generally choose news sources that fit their expectations [8].

This phenomenon of confirmation bias is echoed in another heuristic: conformity, the need to cohere with the beliefs of one’s in-group [14,15]. It is adaptive for agents to conform to the behaviours of others in their niche, in part for the very reasons highlighted above [16]. Conformity limits how much information any given agent has to gather to act appropriately, and the sources sampled from their in-group are generally trusted [17]. This is partly due to the fact that members of an in-group can be most precisely predicted: their behaviours are normed, and expected by the members of the group, in ways that generally benefit its members [18,19]. However, conformity has other benefits as well. Being able to sample from the group entails a continued relationship to other members. This will also enable members to acquire pragmatic resources beyond information (e.g., food and shelter), as the group generally provides for its members [20,21]. Being cut off from the group can lead to existential difficulties [22,23]. Group members can be sanctioned if they fail to conform to the norms, including epistemic norms [24,25].

### 1.2. The Spread of Ideas

These two heuristics—namely, confirmation bias and conformity—mutually reinforce each other. Specifically, to save energy, confirmation bias leads to agents’ being drawn to groups that validate their opinion, and thus increases the probability of behavioural and epistemic conformity [26]. Importantly, these two heuristics form the basis for information spread. Agents spread information through media and through connections to one another, given a network structure [27]. The spread of ideas and behaviours from one agent to another serves both local and larger-scale coordination [27,28].

The spread of ideas is facilitated when agents are already attuned to them. Individuals are more likely to adopt ideas that they believe will have a positive effect on them, especially if the outcome of sharing that information will be positive [29]. According to Falk and Scholz, this entails that sharing among group members of news that dovetails with group norms is likely to lead to the adoption of these ideas among the other group members, following the conformity heuristic mentioned earlier. One way to predict whether information will be coherent with the group norms is to assess it with respect to one’s own value system. Naturally, similar individuals within a given group who share values will be more likely to spread ideas [30,31].

This notion of attunement or synchronisation is fundamental. Synchronisation across network nodes lowers the cost of information flow [32], and increases the certainty of the message being spread, as well as the quality of its reception, even if the message itself may be prone to errors [33,34]. Specifically, a message will be more intelligible to group members who share a common set of codes, and agents are more likely to integrate new information if it fits with their understanding of the world [35,36,37].

Hashtags have been shown to be heavy carriers of information in echo-chambers. They tend to be used in partisan ways, to reach people of similar mindsets, as well as to signal one’s own partisanship affiliation [38]. The spread of information is optimised through hashtags as pseudo-meta-linguistic categorisation makers [39].

### 1.3. Communities Forming around Ideas

Thus, the beliefs and epistemic communities of agents develop together, synchronously. We label communities formed in this process of belief sharing as ‘epistemic communities’. Such communities share and spread a worldview, or a paradigm, and normalise sampling behaviours (i.e., manners of observing and engaging with the shared social world) that reinforce this view of the world [40]. Individuals in the community are tied together by these epistemic practices, further reinforcing the social signals which act as evidence for the shared model of the world [19].

One example of these communities is the *echo-chamber*, a phenomenon that has been studied significantly in social media [38,41,42,43,44,45,46,47]. Echo-chambers are an extreme example of epistemic communities, and they have components that enforce their formation and maintenance [48,49,50]. Echo-chambers tie people with similar views together, and tend to actively work against the engagement with, and assessment and evaluation of, external sources (e.g., information provided by members of the outgroup) [42,48]. Echo-chambers can become epistemically vulnerable when members can no longer assess whether a piece of information is true or not [48,51]. Similarly, only having access to a few sources limits how much information can be gathered, and relevant sources of evidence may fall through the gaps [49,52]. According to [52], error will be propagated, and it will be difficult to check errors against anything, as most minds in the echo-chamber are synchronised and therefore poised to make the same mistakes.

### 1.4. Volatility and Habit Formation

Studies on the perception of environmental volatility range from economics to psycho-education for the autism spectrum [53,54,55]. Optimal inference in a changing world requires integrating incoming sensory data with beliefs about the intrinsic volatility of the environment. Intuitively, environments with higher volatility change more quickly and thus have a shorter intrinsic timescale—and conversely for environments with lower volatility. For example, autistic individuals tend to pay more attention to small changes in the environment, giving them a better ability to track potentially important fluctuations in information [53]. On the other hand, this increased attention to environmental fluctuations may also lead to increased sensitivity to random, non-informative changes in the environment, a phenomenon that might be called (from a signal-detection perspective) a higher ‘false-positive’ rate [53].

When this type of precision dynamics [56] is applied to the social field at large, emergent epistemic phenomena can be explained. For instance, during the COVID-19 pandemic, the certainty around knowledge was very low, as information about the pandemic and the biology of the virus was limited [57,58]. In addition, alternative sources of information (e.g., anti-vaccine conspiracies) had become more prevalent and more influential in some social networks [59]. The gravity of the affliction, and the strength of the governmental response, also made any information on the topic vitally important, and worth one’s attention [60]. This prompted an intensive use of information technology in order for individuals to find answers (“doing one’s own research”). This excessive use points to the awareness by laypeople of the high volatility of the topic. The authors of [61] measured emotional volatility on social media in China during the pandemic, and explored the social dynamics underlying the emotional volatility.

Individuals can deal with volatility by using various coping mechanisms. One such mechanism is to constrain the uncertainty related to their own behaviours via *habit formation* [62,63,64,65,66,67,68,69,70,71]. In this paper, we model habit formation as a form of behavioural reinforcement, where behaviours become more probable as a function of how often they are engaged in [72,73]. If behaviour is initially goal- or information-driven, habit-learning can then ‘zero-in’ and isolate the invariant features of such (initially) goal-directed behaviour [74], mirroring the so-called transition from ‘model-based’ to ‘model-free’ decision-making in reinforcement learning [75,76]. After an agent has engaged in a given behaviour enough, even if that behaviour is initially pursued in a goal-driven manner, a habit can then be formed and become hard to ‘unlearn’ [77]. This view also supports the idea that, initially, habit-formation can be goal-driven. In the current context of echo chamber formation, confirmation bias may serve as the original ‘motivation’ that later underwrites preferential sampling behaviour. In combination with habit learning, it may then become impossible to stop enacting this peer-specific sampling, even in the face of changing information.

### 1.5. An Active Inference Model of Epistemic Communities

This paper introduces a computational model of epistemic communities, wherein individual agents share information with one another and come to form beliefs not only about their local environment, but also about the beliefs of other agents in their community. To understand this phenomenon, we leverage the active inference framework, a first-principles theory of cognition, which explains the manner in which agents select actions based on their causal model or understanding of the world. Active inference says that organisms act to minimise a quantity called variational free energy, which quantifies the divergence between expected and sensed data. From this point of view, to select an action is to infer ‘what I must be doing, given what I believe and what I sense’. Extensive work has been done in the field of active inference to study social systems and the way in which the minimisation of free energy could give rise to (eventually large-scale) behavioural coordination [3,15,78,79,80,81,82,83]. However, much of this work is still theoretical.

At first glance, it might appear difficult to model a phenomenon like confirmation bias using an active inference formulation, because action selection in active inference is guided by the principle of maximising Bayesian surprise or salience, which requires constantly seeking out information that is expected to ‘challenge’ one’s world model [84,85,86].

However, the key notion that allows ‘confirmation bias’ to nonetheless emerge under active inference is ultimately the *subjective* nature of information gain, also known as ‘epistemic value’. Crucially, this Bayesian surprise or information gain term is always an *expected* surprise—that is, what counts as an ‘information-maximising’ observation is always defined in relation to agent’s set of beliefs or generative model. Due to this inherent subjectivity, the true informativeness or epistemic value of an action can be arbitrarily far from the agent’s expectation thereof. Taking advantage of this, in the model presented here, we endow agents with what we refer to as *epistemic confirmation bias*. This is implemented by building a prior belief into the generative model, namely that agents are more likely to sample informative observations from agents with whom they agree a priori. Therefore, agents will sample agents with whom they agree under the (not necessarily true) belief that such agents are more likely to provide higher quality information.

We can make two important distinctions between the kind of polarisation that we observe in traditional opinion dynamics and the kind achieved through multi-agent active inference modelling. First, in traditional approaches, the implementation of bounded confidence to motivate polarisation is essentially a hard-coded restriction on the agents’ ability to perceive and therefore update their beliefs [87,88,89,90]. By contrast, in the active inference approach, polarisation is instead motivated by the positive effect of confirmation bias, which is integrated directly in the agents’ (likelihood) model of the world, which allows agents to acquire more evidence about their environment if the information comes from another agent that shares the same worldview. This means that agents are motivated implicitly in their generative models to gain more evidence about the world if this evidence confirms their preexisting beliefs. Second, in the traditional approaches, agents can directly perceive the ‘belief state’ of other agents, and thus, the opinion of one agent directly influences that of another [87,89]. This is an unrealistic assumption, since human agents have to infer the belief states of others by interpreting their behaviour. This aspect of belief inference is a cornerstone of the active inference approach: the belief of another agent is modelled as a hidden state of the world—thus agents do not have direct access to each others’ belief states. Instead, through inference, they come to hold beliefs about each others’ beliefs, in addition to a belief about some agent-independent ‘world states’ [19,91].

More recently, researchers have begun to build Bayesian models of opinion dynamics, motivated by the Bayesian brain hypothesis and the notion that decision-making is inherently probabilistic [92,93,94,95,96]. Generally, the active inference approach falls within the theoretical umbrella of Bayesian agent-based modelling, because there is a deep assumption that environmental states are inherently hidden (in our case, the belief states of other agents) and need to be inferred on the basis of prior beliefs and sensory observations (i.e., observing the behaviour of other agents). However, as sketched above, a crucial point that distinguishes approaches like active inference and planning as inference from the general Bayesian approach is the notion that *actions themselves are inferred* [97,98] While there have been models that use Bayesian inference for the inference of opinions (i.e., Bayesian belief states about some particular idea), the process of action selection within these works is still often added on after the fact using an arbitrary decision rule (e.g., a softmax function of an arbitrary value vector). Action selection is often cast as a noisy signal of the true belief state, such as in [96], which is then used to update neighbouring agents’ beliefs through Bayesian inference. Crucially, in active inference, behaviour itself is cast as the result of inference, specifically by sampling actions from a posterior distribution over actions. The posterior over actions is obtained by minimising the expected free energy of future beliefs, conditioned on actions. In other words, actions are selected in order to achieve goals and minimise future uncertainty, i.e., to maximise a lower bound on Bayesian model evidence.

Importantly for our purposes, one can supplement this goal-directed aspect of policy inference, driven by the expected free energy, with inflexible ‘prior preferences over actions’, i.e., habits. If this prior preference over actions is learned over time, then in the context of the opinion dynamics model presented here, this can lead to a propensity to continue sampling agents that have been sampled previously. The idea of choosing actions through inference in accordance with the minimisation of uncertainty is powerful as a modelling technique, because through the choice of policy preferences one can encode various social behaviours, such as conformity, habit formation, hostility, or indifference, while in this report, only habit formation, conformity, and polarisation are explored, we emphasise the potential of augmenting the current model to capture a wider range of features observed in human social behaviour.

### 1.6. Hypotheses

In this paper, we present a multi-agent model of opinion dynamics based on the active inference formulation. Our simulated agents are situated in a social network where they observe the behaviour of other agents and update their beliefs about a pair of abstract, mutually exclusive “Ideas” (e.g., the truth values of two competing claims), as well as the beliefs of their neighbours in the social network. Agents themselves have a prior preference to announce their beliefs via an action that is observable by other agents (e.g., posting/tweeting a “hashtag”). We show that the proposed active inference model can replicate confirmation bias, exposure effects, the formation of echo-chambers, and the exacerbation of these phenomena via habit-learning. These effects can be modelled by changing the parameters of individual generative models, i.e., the cognitive features of the individuals comprising the group. We also uncover interesting interactions between individual-level cognitive features and the network architecture that constrains their social interactions. The large-scale behaviour of the model can be used to test three hypotheses, which are motivated by the existing literature. We formulate and test three hypotheses as follows:

**Hypothesis** **1.** *We cast confirmation bias in active inference as a form of ‘biased curiosity,’ in which agents selectively gather information from other agents with whom (they believe) they agree, under the assumption that like-minded agents provide higher-quality, more reliable information. We hypothesise that this ‘epistemic confirmation bias’ can mediate the formation of echo-chambers and polarisation in social networks of active inference agents. However, we further hypothesise that epistemic confirmation bias and network connectivity will bidirectionally modulate the formation of polarised epistemic communities, tuning the collective trade-off between deadlock (polarisation) and agreement (consensus).*

**Hypothesis** **2.** *We also consider the effect of agents’ beliefs about the volatility of their social environments. In particular, we examine how beliefs about social volatility impact exploratory sampling of other agents’ perspectives, which itself may interact with epistemic confirmation bias to determine the formation of echo-chambers. In particular, we hypothesise that beliefs about less quickly changing social environments (a belief in lower social volatility) will increase the likelihood of polarisation, as opposed to consensus.*

**Hypothesis** **3.** *Finally, we also hypothesise that we can model selective exposure effects and conformity through habit formation, which naturally emerges through Bayes-optimal learning of a prior distribution over policies. We hypothesise that a greater learning rate for habit formation will lead to clusters within the network, thus amplifying and quickening the formation of echo-chambers.*

Using the multi-agent active inference model of opinion dynamics, we achieve simulation outcomes that reproduce phenomena observed in the opinion dynamics literature, such as polarisation and consensus. In the sections to follow, we first describe the generative model that each agent uses to engage in active inference, and then discuss how we couple the agents together in an opinion dynamics network. We conclude by presenting numerical results that investigate each of the three hypotheses laid out above.

## 2. An Active Inference Model of Opinion Dynamics

### 2.1. Overview

We present a multi-agent active inference model of opinion dynamics on an idealised social network. In the model, a group of agents simultaneously update their beliefs about an abstract, binary hidden state (that represents two conflicting “Ideas”) and the opinion states about these ideas, held by a limited set of neighbouring agents. Each agent also generates an action that is observable to other agents. In the context of digital social networks like Twitter, these observable actions could be analogised to ‘posts’, ‘tweets’ or ‘hashtags’, i.e., some abstract expression carrying information about the belief state of the agent generating that expression. Hereafter we refer to these actions as ‘tweeting a Hashtag’ and describe agents‘ behaviour as the decision to ‘tweet Hashtag 1 vs. Hashtag 2’, etc. Over time, each agent updates a posterior distribution (or belief) about which of the two Ideas is true, as well as a belief about what a connected set of other agents in the network believe (namely, those agents whom they ‘follow‘ or are ‘followed by’ in the social network). Both of these inferences are achieved by observing the behaviour of other agents, where, crucially, this behaviour depends on each agent’s beliefs (notably about other agents). In our formulation, agents can only observe the behaviour of other agents to whom they are specifically connected.

It is worth emphasising that in this formulation, there is no *true* hidden state that corresponds to the competing truth status of the two “Ideas.” Rather, this abstract binary hidden state is only contained in the *generative model* or internal representation of each agent. The only ‘real’ states of the system are the agents and their observable behaviour.

In the sections to follow, we first briefly summarise the previous literature on computational approaches to the study of opinion dynamics. We then review the formalism of active inference, from the specification of the generative models that each agent uses to represent their external world, to the update equations for state estimation and decision-making. Finally, we describe the simulations of multi-agent dynamics by linking an ensemble of such active inference agents into a network.

### 2.2. Opinion Dynamics Models

In previous models of opinion dynamics, individual agents are often characterised by one or a few variables that encode the current belief or opinion held by that agent [99,100,101]. Collections of agents then update their respective opinion variables by ‘observing’ other variables that (either deterministically or stochastically) depend on the opinions of other agents in the ensemble. The nature of the inter-agent interactions varies across different models, ranging from homogeneous, ‘mean-field’-like global potentials [102,103] to structured, heterogeneous networks with fixed or dynamic weights between agents [104,105]. The opinion variables can take scalar or vector-values [106,107], and have either discrete or continuous support [108,109,110,111].

Bayesian variants of opinion dynamics models explicitly take into account the *uncertainty* associated with the observations and decisions of agents, where now the updates to opinion variables become (exact or approximate) Bayesian updates [95,96,112,113]. The active inference model we present here is an example of such a Bayesian approach, with a few crucial distinctions, such as the approximate (as opposed to exact) nature of the Bayesian belief updating, and the fact that actions, in addition to opinions, are the result of inference. We will detail these distinctions further in the sections below on active inference.

### 2.3. Active Inference

Active inference is a biologically motivated framework that rests on first principles of self-organisation in complex, adaptive systems [86,97,114]. Particularly, it is premised on the notion that the internal states of any biological system are statistically insulated from the environment that generates sensory observations, and thus must engage in inference (about the causes of its sensory states) to behave optimally [115]. Active inference finesses this fundamental uncertainty by adding a Bayesian twist, proposing that biological systems entertain or entail a generative model of the latent environmental causes of their sensory inputs. Therefore, unlike classic reinforcement learning or reflexive behavioural algorithms (e.g., state-action policy mapping [72,116]), actions taken under active inference are guided by internal beliefs, which themselves are optimised with respect to an internal ‘world model,’ or representation of the world’s causal and data-generating structure.

Crucially, active inference agents represent their own actions (and their typical sensory consequences) in their generative model. By performing inference with respect to both hidden environment states of the world and the consequences of their own actions, active inference agents can evince behaviour that both (1) achieves their goals or fulfils preferences and (2) actively reduces uncertainty in the agent’s world model [86,97,115]. An active inference agent’s only imperative is to increase model evidence, or equivalently, to *reduce surprise*. Processes such as learning, perception, planning, and goal-directed behaviour emerge from this single drive to increase evidence for the agent’s generative model of the world.

In active inference, the agents never act directly on sensory data, but rather change their beliefs about what causes that data. Thus, the core step in active inference consists of optimising these beliefs using a generative model. This process is also known as Bayesian inference or Bayesian model inversion. Inference answers the question: “what is my best guess about the state of the world, given my sensory data and prior beliefs”? This can be formalised using Bayes’ rule:

(1) $$\begin{array}{c} P\left(\vartheta |y\right)=\frac{P(y|\vartheta )P(\vartheta )}{\sum_{\vartheta }P\left(y|\vartheta \right)P\left(\vartheta \right)} \end{array}$$

where the optimal belief about ‘hidden’ or latent variables ϑ, given some sensory data *y*, is called the *posterior distribution*P(ϑ|y). Bayes’ rule yields an analytic relationship between the generative model P(y,ϑ) and the posterior. Bayesian inference consists in calculating (either analytically or approximately) P(ϑ|y). Active inference is no different: perception (the generation of a best guess about the current hidden states of the world) is formalised as the computation of a posterior distribution over hidden states *s*, and action (the *active* part of active inference) is formalised as the computation of a posterior distribution over policies π. In active inference, however, this problem is turned into one of *approximate* Bayesian inference, where instead of finding the optimal posterior P(s|o), active inference agents instead approximate this optimal posterior with a variational posterior Q(s;ϕ), i.e., a belief over hidden states that is parameterised by variational parameters ϕ. The reason for this is that the exact inference is often computationally intractable. The marginalisation problem involved in exact Bayesian inference (expressed in Equation (1)) is often intractable for many realistic generative models. Variational inference turns this intractable calculation of the marginal into an optimisation problem, where a variational upper bound on *surprise* known as variational free energy (also known as negative model evidence in statistics) is minimised:

(2) $$\begin{array}{cc} Q^{*}\left(s;\phi \right) & =\underset{\phi }{\mathrm{argmin}}\underset{\mathrm{surprise}\;\mathrm{bound}}{\underset{︸}{D_{KL}\left(Q(s;\phi )\|P(o,s)\right)}}\\ D_{KL}\left(Q(s;\phi )\|P(o,s)\right) & =D_{KL}\left(Q(s;\phi )\|P(s|o)\right)\underset{\mathrm{surprise}}{\underset{︸}{-\log P(o)}} \end{array}$$

where $D_{KL}\left(q\|p\right)$ is the Kullback–Leibler divergence, a non-negative measure of difference between probability distributions, where $D_{KL}\left(q\|p\right)=0$ when q=p. Variational inference thus consists of optimising the variational parameters ϕ in order to minimise the free energy, which itself renders the variational posterior a better approximation to the true posterior. When variational inference is exact, the bound becomes exact and the free energy reduces to the surprise or negative log evidence. The remaining (negative) surprise can be itself used as a score for model averaging and model selection [117,118].

Active inference agents achieve perception and action by minimising the surprise bound in Equation (2) with respect to variational beliefs about particular variables of their generative model. Optimising beliefs about variables that represent latent environmental states (often denoted *s*) is proposed as a formal model of perception, while optimising beliefs about variables that correspond to policies or control of the environment (often denoted by *u* or π) is the formal analogue of planning and action. Therefore, active inference agents infer both hidden states (perception) and policies (action) through a process of variational inference. The update equations used for perception and planning under active inference are detailed in Section 2.10, Section 2.11 and Section 2.12.

Specifying a generative model P(o,s) is critical to determining the behaviour of active inference agents. In the following sections we introduce the discrete state space model, a partially observed Markov decision process or POMDP, with which we equip agents in the multi-agent opinion dynamics setting.

### 2.4. Generative Model

Formally, the generative model is a joint probability distribution P(o,φ) over observations *o* and latent variables φ. Intuitively, one can think of the generative model as the agent’s ‘representation’ of its environment, and specifically how that environment elicits observations [119]. In the discrete generative model described below, this generative model comprises assumptions about how hidden states s and actions u are probabilistically related to one another and to observations o.

In the current study, agents entertain *partially observed Markov decision process* generative models, or POMDPs [120,121]. POMDPs are a class of decision-making models commonly used to simulate planning and decision-making in environments where agents must at each timestep select one of a discrete set of mutually exclusive options. This is often represented using several random variables: a discrete set of actions *u* (also known as control states); hidden states *s*, which evolve according to (action-dependent) Markovian dynamics; and observations *o*, which probabilistically depend upon current hidden states. In most active inference models using POMDP generative models, hidden states, observations, and actions are discrete random variables—namely, they can take one of a finite set of values at a given time.

We include an additional latent variable, policies π, in the generative model. Policies are simply sequences of control states *u*. Using the terminology above, our generative model can be written down as $P(\tilde{o},\tilde{\varphi })$ where $\tilde{\varphi }=\left\{\tilde{s},\tilde{u},\pi \right\}$. The tilde notation $\tilde{x}$ denotes a sequence of random variables over time, e.g., $\tilde{s}=s_{1,\ldots ,T}$.

We can now write down the Markovian generative model as follows:

(3) $$\begin{array}{cc} P\left(\tilde{o},\tilde{s},\tilde{u},\pi \right) & =P\left(s_{1}\right)P\left(u_{1}\right)P\left(\pi \right)\prod_{\tau =2}^{T}P\left(s_{\tau }|s_{\tau -1},u_{\tau }\right)P\left(u_{\tau }|\pi \right)\prod_{\tau =1}^{T}P\left(o_{\tau }|s_{\tau }\right) \end{array}$$

The observation likelihood $P(o_{\tau }|s_{\tau })$ represents the agent’s probabilistic understanding of the relationship between hidden states $s_{\tau }$ and concurrent observations $o_{\tau }$. Because both observations $\tilde{o}$ and states $\tilde{s}$ are discrete, this likelihood distribution will be represented as a multidimensional array, which we hereafter denote by A. Similarly, the transition distributions $P(s_{\tau }|s_{\tau -1},u_{\tau })$, which are denoted by B, encode the agent’s beliefs about how hidden states and control states determine subsequent hidden states. It is by changing actions $u_{\tau }$ that the agent can exert control on its environment, since the evolution of hidden states depends both on the past state $s_{\tau -1}$ and on the concurrent action $u_{\tau }$. Finally, the distribution $P(u_{\tau }|\pi_{\tau })$ represents the mapping between policies and actions.

In many POMDP models, we segregate observations $\tilde{o}$ and hidden states (and controls) $\tilde{s}$ (resp. $\tilde{u}$) into distinct *modalities* (for observations) and *factors* (for hidden states/control states):

(4) $$\begin{array}{c} \tilde{o}=\left\{{\tilde{o}}^{\left(1\right)},{\tilde{o}}^{\left(2\right)},\ldots ,{\tilde{o}}^{\left(M\right)}\right\}\;\tilde{s}=\left\{{\tilde{s}}^{\left(1\right)},{\tilde{s}}^{\left(2\right)},\ldots ,{\tilde{s}}^{\left(F\right)}\right\}\;\tilde{u}=\left\{{\tilde{u}}^{\left(1\right)},{\tilde{u}}^{\left(2\right)},\ldots ,{\tilde{u}}^{\left(F\right)}\right\} \end{array}$$

where the superscripts refer to the index of the modality or factor index, respectively.

Observation modalities can be thought of as sensory ‘channels’ that provide distinct sorts of information. For example, in the context of human cognition, observation modalities might correspond to the information originating in different sense organs, e.g., the ears, eyes, or skin.

Hidden state factors may be thought of as the generative model’s latent representation of different features of the external world. Each of these factors has its own dynamics and can be thought of as statistically independent of other factors. For instance, an object might be described by both its spatial location and its colour—‘location’ and ‘colour’ would thus be candidates for distinct hidden state factors in a generative model of an object. This factorisation is motivated by our intuition that something like an object’s colour and location are independent. An additional, minor note is that control states (the agent’s representation of its own actions or ability to intervene on hidden states) are also divided into a set of control factors, with one control factor for every hidden state factor.

Given this factorisation, at any given time a single observation will thus comprise a *set* of modality-specific observations, one from each sensory channel, and a hidden state will comprise of a *set* of hidden states, one from each distinct hidden state factor.

Now that we have introduced the class of discrete generative models with which our active inference agents will be endowed, we are now in a position to articulate the particular structure of the generative model for a single agent. From here, using active inference to perform inference and action with respect to each single agent’s generative model, we can then ‘link together’ ensembles of these agents to form a complete opinion dynamics simulation.

### 2.5. An Individual Model of Opinion Formation

**Figure 1:**
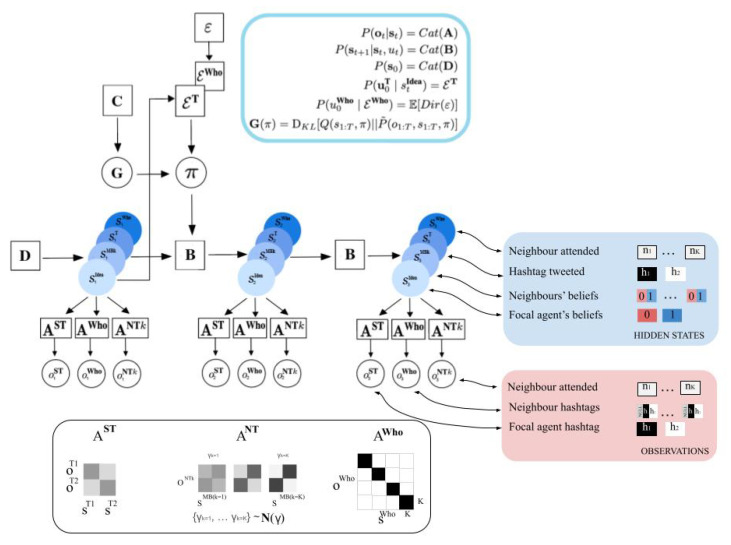
Bayesian network representation of the POMDP generative model. Squares represent priors, likelihoods, or ‘factors’ that relate random variables to one another, and circles represent random variables (stochastic nodes). Different hidden state factors are represented as state variables and the different modality-specific $A^{\left(m\right)}$ arrays of the observation model shown are side by side, since they lead independently to the observations generated in that modality, but are conjunctively dependent on hidden state factors. Note that the B array can be similarly decomposed into different sub-arrays, one per hidden state factor, but it is shown as a single square here for simplicity. The prior over policies is parameterised by E, which has separate prior over control states ($E^{\mathrm{Who}}$ and $E^{T}$) for each control state factor. The box at the top right contains mathematical descriptions of each component in the generative mode. Note that while it is included in the graphical model, we left out the C vector since it is not relevant for the current model.

We describe a generative model of opinion formation for a single agent. Note that each active inference agent in the multi-agent simulations described below will be equipped with this same basic generative model. A single agent (hereafter: the ‘focal agent’) observes the actions of other agents, forms beliefs about an abstract binary environmental state, and chooses actions, which themselves are observable to other agents. The focal agent’s action consists of two simultaneous choices: an ‘expression’ action (choosing which observable expression to make) and an ‘observation’ action (choosing which other agent to attend to). As mentioned above, we analogise the ‘expression’ actions to posts made by users on online social networks (e.g., ‘tweets’, ‘re-tweets’, ‘shares’, ‘likes’), and the contents of these actions we refer to as ‘Hashtags.’ Crucially, an agent can only observe one neighbouring agent at a time. Therefore, at each timestep, a focal agent both tweets its own Hashtag and chooses to read the Hashtag tweeted by another single agent. See Figure 1 and Table 1 for a summary of the distributions and random variables that comprise a single agent’s generative model of opinion formation.

#### 2.5.1. Hidden States

Each agent’s generative model comprises hidden states that fall into four categories—however, the actual number of hidden state factors per agent depends on their local network connectivity, so that a particular agent will usually have *more than* four hidden state factors. Nevertheless, we classify each hidden state factor into one of these four categories:

1. $s^{\mathrm{Idea}}$: A binary random variable that encodes the agent’s beliefs about an abstract environmental state that represents the truth value of two mutually exclusive Ideas or claims. This binary variable can thus take a value of either 0 or 1, to which we assign arbitrary labels of **Idea 1** and **Idea 2**. If **Idea 1** is true, then necessarily **Idea 2** is false, and vice versa.
2. $s^{\mathrm{MetaBelief}}$ (shortened to: $s^{\mathrm{MB}}$): A set of binary random variables, each of which corresponds to a *particular neighbour’s belief* about which of the two Ideas is true. As a representation of another agent’s belief, we hereafter refer to this class of hidden state factor (and corresponding posteriors) as ‘meta-beliefs’. The values of this variable we label **Believe Idea 1** and **Believe Idea 2**. Each agent will have one hidden state factor belonging to this category for each of its *K* neighbours, e.g., $s^{\mathrm{MB}1},s^{\mathrm{MB}2},\ldots ,s^{\mathrm{MB}K}$.
3. $s^{\mathrm{SelfTweet}}$ (shortened to: $s^{T}$): A binary random variable corresponding to what the focal agent is currently doing. By analogy with Twitter and other digital social media platforms, we refer to this action as ‘tweeting‘ or ‘posting’, and the variable can take a value of either 0 or 1, representing one of two possible contents (’Hashtags’). These two actions are thus labeled **Tweet Hashtag 1** ($s^{\mathrm{SelfTweet}}=0$) and **Tweet Hashtag 2** ($s^{\mathrm{SelfTweet}}=1$).
4. $s^{\mathrm{WhoAttend}}$ (shortened to: $s^{\mathrm{Who}}$): A multinomial random variable with as many discrete levels as the focal agent has neighbours, representing which of their neighbours’ actions the focal agent is currently attending to. For example, for an agent with three neighbours, this variable could take three values: (0,1,2), which we label **Attend Neighbour 1**, **Attend Neighbour 2**, and **Attend Neighbour 3**, respectively.

For a single agent’s generative model, the precise *number* of ‘meta-belief’ hidden state factors (those belonging to the $s^{\mathrm{MB}}$ class of factors) depends on how many neighbours the focal agent has. For instance, if a given agent *i* has three neighbours, then that agent’s generative model will have three meta-belief hidden state factors: $s^{\mathrm{MB}1}$, $s^{\mathrm{MB}2}$, and $s^{\mathrm{MB}3}$, each representing the belief state of one of agent *i*’s three neighbours. Each agent has only *one* hidden state factor belonging to the other categories: $s^{\mathrm{Idea}}$, $s^{T}$, and $s^{\mathrm{Who}}$. However, the *cardinality* (i.e., number of levels) for the $s^{\mathrm{Who}}$ hidden state factor will be equal to the focal agent’s number of neighbours. In the case of our agent *i* with three neighbours, therefore, the possible values of $s^{\mathrm{Who}}$ will be (0,1,2), corresponding to the action of attending to one of the three neighbours.

#### 2.5.2. Control States

Each agent is also equipped with two *control state* factors. These state factors are the agent’s representation of its own actions in the environment. Control factors interact with hidden state factors to determine the next hidden state—thus, certain hidden state factors are deemed ‘controllable’ if they are paired with a control factor. In the current model, these two control state factors are paired with hidden state factors in Categories 3 and 4 above:

1. $u^{T}$: A binary random variable corresponding to which ‘tweet action’ to take, i.e., **Tweet Hashtag 1** vs. **Tweet Hashtag 2**. This control factor interacts with the $s^{T}$ hidden state factor.
2. $u^{\mathrm{Who}}$: A multinomial random variable corresponding to which neighbour to attend to, e.g., **Attend Neighbour 1**, **Attend Neighbour 2**, and **Attend Neighbour 3**. This control factor interacts with the $s^{\mathrm{Who}}$ hidden state factor.

#### 2.5.3. Observation Modalities

Just as we did for the hidden states, now we describe three categories of observation modalities for a single agent’s generative model:

1. $o^{\mathrm{SelfTweet}}$ or $o^{\mathrm{ST}}$: A binary random variable representing the focal agent’s observation of its own tweet actions—these ‘self-observations’ take the values of **Hashtag 1** and **Hashtag 2**.
2. $o^{\mathrm{NeighbourTweet}}$ or $o^{\mathrm{NT}}$: A ternary random variable representing the observation of a neighbour agent’s actions—these take the values of **Null**, **Hashtag 1**, and **Hashtag 2**. Each agent has one ‘tweet observation’ modality for each of its *K* neighbours: $o^{\mathrm{NT}1},o^{\mathrm{NT}2},o^{\mathrm{NT}3},\ldots ,o^{\mathrm{NT}K}$, in the same way that the number of $s^{\mathrm{MB}}$ factors depends on the number of neighbours. The purpose of the **Null** observation level will be clarified later on.
3. $o^{\mathrm{WhoAttend}}$ or $o^{\mathrm{Who}}$: A multinomial random variable representing the observation of which neighbour the focal agent is attending to. This random variable has as many discrete levels as the focal agent has neighbours. For example, for an agent with three neighbours, this variable could take three values: (0,1,2), which we label **Attend Neighbour 1**, **Attend Neighbour 2**, and **Attend Neighbour 3**.

A focal agent receives a full multi-modality observation per timestep:

(5) $$\begin{array}{cc} o_{t} & =\{o_{t}^{\mathrm{ST}},o_{t}^{\mathrm{NT}1},o_{t}^{\mathrm{NT}2},\ldots ,o_{t}^{\mathrm{NT}K},o_{t}^{\mathrm{Who}}\} \end{array}$$

Each single observation is thus a collection of observations, one from each modality. Because one observation is collected from each modality at every timestep, the cardinality of some modalities is increased by 1, creating an additional observation level which we can call the “**Null**” observation level. The **Null** observation is included to effectively ‘block’ the focal agent from seeing the Hashtags of neighbours they are not actively attending to. This observation level is designed to have maximal ambiguity with respect to hidden states—in other words, seeing a **Null** observation affords no information about hidden states and thus has no effect on inference. This will become more clear when the observation and transition likelihoods of the generative model are described.

### 2.6. Likelihoods

Having specified the random variables that form the support of a single agent’s POMDP generative model, we can now move onto describing the likelihoods that determine how hidden states relate to observations, and how hidden states relate to each other over time. The construction of these likelihoods is indispensable for understanding both the belief updating and the choice behaviour of active inference agents.

We begin with the observation likelihood model $P(o_{t}|s_{t})$. This is also known as the ‘sensory likelihood’ or observation model, and is parameterised by a series of categorical distributions whose parameters we collectively encode as the columns of a multidimensional array called A. In other words:

$$\begin{array}{c} P\left(o_{t}|s_{t}\right)=Cat\left(A\right) \end{array}$$

The entire A array is actually a set of tensors, with one sub-tensor per observation modality:

$$\begin{array}{c} A=\{A^{\mathrm{ST}},A^{\mathrm{NT}1},A^{\mathrm{NT}2},\ldots ,A^{\mathrm{NT}K},A^{\mathrm{Who}}\} \end{array}$$

Each modality-specific likelihood tensor $A^{m}$ is a potentially multidimensional array that encodes the conditional dependencies between each combination of hidden states $s_{t}=\left\{s_{t}^{1},s_{t}^{2},\ldots ,s_{t}^{F}\right\}$ and observations $o_{t}^{m}$ for that modality. For example, in a likelihood array with two hidden state factors, entry ${\left[A^{m}\right]}_{ijk}$ encodes the conditional probability $P(o_{t}^{m}=i|s_{t}^{1}=j,s_{t}^{2}=k)$, i.e., with the probability of observing outcome *i* within observation modality *m* under hidden state factor 1 being level *j* and hidden state factor 2 being level *k*. In the case of the generative model for opinion formation, these likelihood arrays will be of much higher dimensions than 3-D tensors; we will thus generally refer to the elements of a modality-specific $A^{m}$ array with the notation ${\left[A^{m}\right]}_{ijk\ldots }$, where the ellipses refer to an indefinite number of indexable lagging dimensions.

Each agent in the opinion dynamic model will have one $A^{m}$ array per observation modality. We will now step through them to describe their role in the generative model.

#### 2.6.1. Self Tweet Likelihood

The array $A^{\mathrm{ST}}$ represents the agent’s beliefs about how hidden states relate to $o^{\mathrm{ST}}$ (which content the agent is tweeting, either **Hashtag 1** or **Hashtag 2**). By construction, $A^{\mathrm{ST}}$ encodes an assumption that $o^{\mathrm{ST}}$ only depends on $s^{T}$, the controllable hidden state factor corresponding to the tweet action. This is an unambiguous or isomorphic mapping, which we can express as follows:

(6) $$\begin{array}{cc} A^{\mathrm{ST}} & =P\left(o_{t}^{\mathrm{ST}}|s_{t}^{T}\right)=I_{2}=\left[\begin{array}{cc} 1 & 0\\ 0 & 1 \end{array}\right] \end{array}$$

In other words, the agent believes that the $s^{T}$ factor unambiguously signals its true value via the $o^{\mathrm{ST}}$ observation modality. Each column of the matrix in Equation (6) represents a (conditioning) value of $s^{T}$, and each row represents a (conditioned) value of $o^{\mathrm{ST}}$. The value of $o^{\mathrm{ST}}$ does not depend on any of the other hidden state factors, which means that this identity matrix is uniformly ‘tiled’ across the other dimensions of the $A^{\mathrm{ST}}$ array that represent the mapping between the remaining hidden state factors $\left\{s^{\left(1\right)},s^{\left(2\right)},\ldots \right\}\notin s^{T}$ and $o^{\mathrm{ST}}$.

#### 2.6.2. Neighbour Tweet Likelihood

The array $A^{\mathrm{NT}k}$ represents the focal agent’s beliefs about how hidden states relate to $o^{\mathrm{NT}k}$, the focal agent’s observation of neighbour *k*’s tweet content. $A^{\mathrm{NT}k}$ encodes an assumption that $o^{\mathrm{NT}k}$ probabilistically depends on neighbour *k*’s belief about the two Ideas, i.e., that $o^{\mathrm{NT}k}$ depends on $s^{\mathrm{MB}k}$. This can be expressed as:

(7) $$\begin{array}{cc} A^{\mathrm{NT}k} & =P\left(o_{t}^{\mathrm{NT}k}|s_{t}^{\mathrm{MB}k},s_{t}^{\mathrm{Who}}=k\right)=\left[\begin{array}{c} 0\\ h \end{array}\right] \end{array}$$

where 0 represents a 1×2 vector of 0s, and h is a 2×2 matrix that represents the ‘Hashtag semantics,’ i.e., the assumed relationship between neighbour *k*’s beliefs and what Hashtag they are expected to tweet. Importantly, the first row of the likelihood matrix in Equation (7) represents the probability of encountering the **Null** observation, for the various settings of hidden states. This observation always has probability 0 when the focal agent is sampling neighbour *k*, as represented by the condition $s_{t}^{\mathrm{Who}}=k$. Otherwise, when $s_{t}^{\mathrm{Who}}\neq k$, the **Null** value will be expected with certainty. This can be expressed as:

(8) $$\begin{array}{cc} A^{\mathrm{NT}k} & =P\left(o_{t}^{\mathrm{NT}k}|s_{t}^{\mathrm{MB}k},s_{t}^{\mathrm{Who}}\neq k\right)=\left[\begin{array}{cc} 1 & 1\\ 0 & 0\\ 0 & 0 \end{array}\right] \end{array}$$

This inclusion of the **Null** is necessary to ensure that a focal agent only expects to read one of neighbour *k*’s tweet, if they are actively attending to neighbour *k*—otherwise, they receive a ‘blank’ observation that affords no information about hidden states (as represented by a maximally ambiguous likelihood over hidden states, i.e., a row of 1s). The lower two rows of the likelihood matrix in Equation (7) are occupied by the Hashtag semantics h, which we stipulatively define with a ‘Hashtag reliability’ parameter $p_{h}$:

(9) $$\begin{array}{cc} h & =\left[\begin{array}{cc} p_{h} & 1-p_{h}\\ 1-p_{h} & p_{h} \end{array}\right] \end{array}$$

Here, $p_{h}$ parameterises two Bernoulli distributions that, respectively, map between the two levels of $s^{\mathrm{Idea}}$ and the two levels of $o^{\mathrm{NT}k}$. In the limiting case of $p_{h}=1$, this means that the focal agent believes that neighbour *i*’s tweet content is unambiguous evidence for what Idea neighbour *k* believes in. On the other hand, as $p_{h}\to 0$, h comes to resemble a maximum entropy distribution, in this case, according to the focal agent’s generative model, neighbour *k*’s tweet activity provides no information about its beliefs.

This basic conditional relationship outlined in Equations (7)–(9) enables agents to update their beliefs about the beliefs of their neighbours $s^{\mathrm{MB}}$ according what they observe their neighbours tweeting. Intuitively, this mapping captures the focal agents’ beliefs that what their neighbours tweet is representative of what they believe. The accuracy of this mapping (the value of $p_{h}$) determines how strongly Hashtags reflect opinions or the strength of beliefs. However, in order to allow agents to update their beliefs about the truth-values of the Ideas per se (i.e., update a posterior distribution over $s^{\mathrm{Idea}}$), we also construct $A^{\mathrm{NT}k}$ such that agents believe that the validity or truth-values of the Ideas themselves $s^{\mathrm{Idea}}$ probabilistically relate to $o^{\mathrm{NT}k}$. Importantly, we make this conditional relationship ‘biased’ in the sense that, according to $A^{\mathrm{NT}k}$, tweet observations are more precisely related to a particular setting of the $s^{\mathrm{MB}k}$ factor, if and only if $s^{\mathrm{Idea}}$ is aligned with that belief, i.e., when $s^{\mathrm{MB}k}=s^{\mathrm{Idea}}$. This can be formalised as an increased precision γ for subsets of those conditional distributions encoded by $P(o_{t}^{\mathrm{NT}k}|s_{t}^{\mathrm{MB}k},s^{\mathrm{Idea}})$, importantly those subsets when $s_{t}^{\mathrm{MB}k}=s^{\mathrm{Idea}}$. As we will describe later, in the context of action, this leads to an ‘epistemic’ drive for the focal agent to attend to neighbours who (are believed to) share their opinions, leading to a *confirmation bias* effect. We therefore refer to this ‘biased precision’ γ as the *epistemic confirmation bias* (ECB).

(10) $$\begin{array}{c} P\left(o_{t}^{\mathrm{NT}k}=i|s_{t}^{\mathrm{MB}k}=j,s^{\mathrm{Idea}}=j,s_{t}^{\mathrm{Who}}=k,\gamma \right)=\frac{e^{\gamma h_{ij}}}{\sum_{l}e^{\gamma h_{lj}}} \end{array}$$

Note that this additional precision term γ exponentiates the Hashtag semantics matrix h, which is already parameterised by the ‘Hashtag reliability’ parameter $p_{h}$. In the context of inference, an increasing value of γ means that the focal agent believes that tweet observations $o_{t}^{\mathrm{NT}k}$ will provide more information about hidden states, only in the case that the neighbour *k* generating that tweet has ‘correct’ beliefs, i.e., their beliefs are aligned with the true Idea. In the context of decision-making, this means that agents believe that most informative observations come from those neighbours that have the ‘correct’ beliefs. Under active inference, actions that evince informative observations (i.e., observations that resolve the most uncertainty) are preferred. This drive is known as the ‘epistemic value’ or ‘salience’ [86]. Therefore, higher levels of γ will lead to increased epistemic value associated with sampling only those neighbours that the focal agent believes have veridical beliefs, according to its own beliefs about $s^{\mathrm{Idea}}$.

#### 2.6.3. Neighbour Attend Likelihood

The array $A^{\mathrm{Who}}$ represents the agent’s beliefs about how hidden states relate to $o^{\mathrm{Who}}$. This observation model is constructed such that $o^{\mathrm{Who}}$ only depends on $s^{\mathrm{Who}}$, and specifically that agents can always unambiguously infer who they are currently attending to, based on $o^{\mathrm{Who}}$. This can be expressed succinctly as a *K*-dimensional identity matrix:

(11) $$\begin{array}{cc} A^{\mathrm{Who}} & =P\left(o_{t}^{\mathrm{Who}}|s_{t}^{\mathrm{Who}}\right)=I_{K} \end{array}$$

where *K* is the number of the focal agent’s neighbours. Since the value of $o^{\mathrm{Who}}$ does not depend on any hidden state factors besides $s^{\mathrm{Who}}$, $I_{K}$ is ‘tiled’ across the remaining dimensions of the $A^{\mathrm{Who}}$ array.

#### 2.6.4. Transition Model

Now we move onto the transition likelihood model $P(s_{t}|s_{t-1},u_{t-1})$. This is also known as the ‘dynamical likelihood’ and it is parameterised by a series of categorical distributions whose parameters are stored in a tensor *B*:

$$\begin{array}{c} P\left(s_{t}|s_{t-1}\right)=\mathrm{Cat}\left(B\right) \end{array}$$

As there are multiple hidden state factors in our generative model, the full B array is actually split into a collection of sub-arrays, one for each hidden state factor:

$$\begin{array}{c} B=\{B^{\mathrm{Idea}},B^{\mathrm{MB}1},B^{\mathrm{MB}2},\ldots ,B^{\mathrm{MB}K},B^{T},B^{\mathrm{Who}}\} \end{array}$$

Each sub-array $B^{f}$ contains the categorical parameters of the factor-specific transition likelihood $P(s_{t}^{f}|s_{t-1}^{f}|u_{t-1}^{f})$. Note that this construction means that hidden state factors are assumed to be independent by the generative model. In the context of the opinion dynamics model, this means that a single agent assumes that the hidden state $s^{\mathrm{Idea}}$ both does not affect and is not affected by the belief states of neighbouring agents $s^{\mathrm{MB}k}$, and furthermore that the belief states of neighbours do not affect one another. In the following sections, we summarise the transition models for each hidden state factor.

#### 2.6.5. Environmental Dynamics and Volatility

The dynamics of $s^{\mathrm{Idea}}$ according a focal agent’s generative model are described by $B^{\mathrm{Idea}}$. Since this is an uncontrollable hidden state factor, it can be expressed as a simple 2×2 matrix, which expresses the focal agent’s beliefs about the probability that $s^{\mathrm{Idea}}$ (which Idea is “true”) switches over time. We parameterise this matrix with a precision parameter that we call ‘inverse environmental volatility’ $\omega^{\mathrm{Idea}}$:

(12) $$\begin{array}{cc} B^{\mathrm{Idea}} & =P\left(s_{t}^{\mathrm{Idea}}=i|s_{t-1}^{\mathrm{Idea}}=j,\omega^{\mathrm{Idea}}\right)=\frac{e^{{\omega^{\mathrm{Idea}}I}_{ij}}}{\sum_{l}e^{{\omega^{\mathrm{Idea}}I}_{lj}}} \end{array}$$

where *I* is the 2×2 identity matrix. The higher the value of $\omega^{\mathrm{Idea}}$, the more the focal agent believes that the same Idea remains valid over time (e.g., **Idea 1** is likely to remain the ‘valid’ idea from one timestep to the next). Consequently, a lower value of $\omega^{\mathrm{Idea}}$ (and thus a higher value of ‘environmental volatility’) means that the focal agent believes that the truth value of the two Ideas changes less predictably over time (the hidden state is likely to oscillate between Idea1 and Idea2).

#### 2.6.6. Meta-Belief Dynamics and Volatility

The dynamics of $s^{\mathrm{MB}k}$, or the meta-belief associated with neighbour *k* according to a focal agent’s generative model, is described by $B^{\mathrm{MB}k}$. Like $s^{\mathrm{Idea}}$, $s^{\mathrm{MB}k}$ is an an uncontrollable hidden state factor, and the $B^{\mathrm{MB}k}$ array can thus be expressed as a 2×2 matrix. Like $B^{\mathrm{Idea}}$, we parameterise $B^{\mathrm{MB}k}$ with a precision parameter that we term ‘inverse social volatility’ $\omega^{\mathrm{Soc}}$:

(13) $$\begin{array}{cc} B^{\mathrm{MB}k} & =P\left(s_{t}^{\mathrm{MB}k}=i|s_{t-1}^{\mathrm{MB}k}=j,\omega^{\mathrm{Soc}}\right)=\frac{e^{{\omega^{\mathrm{Soc}}I}_{ij}}}{\sum_{l}e^{{\omega^{\mathrm{Soc}}I}_{lj}}} \end{array}$$

The interpretation of $\omega^{\mathrm{Soc}}$ is similar to that of $\omega^{\mathrm{Idea}}$: a higher value of $\omega^{\mathrm{Soc}}$ implies that the focal agent assumes that its neighbours have ‘stubborn’ opinions and are not likely to change over time. A lower value means that the focal agent assumes that its neighbours‘ opinions can easily change over time, or that its neighbours are ‘fickle’.

#### 2.6.7. Tweet Control

Now we discuss the controllable dynamics of the hidden state factor corresponding to the Hashtag that the focal agent is tweeting: $s^{\mathrm{SelfTweet}}$ or $s^{T}$. Under the focal agent’s generative model, this factor only depends on the control state factor $u^{T}$, and the corresponding $B^{T}$ array can thus be expressed as an identity matrix that maps from the action (**Tweet Hashtag 1** vs. **Tweet Hashtag 1**) at timestep t−1 to the next tweet value at timestep *t*:

(14) $$\begin{array}{cc} B^{T} & =P\left(s_{t}^{T}|u_{t-1}^{T}\right)=I_{2}=\left[\begin{array}{cc} 1 & 0\\ 0 & 1 \end{array}\right] \end{array}$$

This means that the agent can unambiguously determine what it tweets next (the value of $s_{t+1}^{T}$) by means of actions $u_{t}^{T}$.

#### 2.6.8. Neighbour Attendance Control

Similarly for the dynamics of $s^{\mathrm{Who}}$, under the focal agent’s generative model, this factor only depends on the control state factor $u^{\mathrm{Who}}$, and the corresponding $B^{\mathrm{Who}}$ array can thus be expressed as an identity matrix that maps from the action of which of *K* neighbours to attend to at timestep t−1, to the next value of $s^{\mathrm{Who}}$ at timestep *t*, namely which neighbour is being attended to:

(15) $$\begin{array}{cc} B^{\mathrm{Who}} & =P\left(s_{t}^{\mathrm{Who}}|u_{t-1}^{\mathrm{Who}}\right)=I_{K} \end{array}$$

Just like the dynamics of $s^{T}$, $s^{\mathrm{Who}}$ is thus fully controllable by the agent, i.e., determined by the value of $u^{\mathrm{Who}}$.

### 2.7. Priors

The next component of the generative model is the *priors* over observations P(o), hidden states $P(s_{0})$, and actions P(u). In discrete active inference models, we represent these as vectors C, D, and E, respectively.

#### 2.7.1. Observation Prior C

In active inference, goal-directed action is often motivated by appealing to a baseline prior over observations P(o|C) that specifies the agent’s preferences to encounter particular outcomes over others. This caches out value in terms of log probabilities or information, rather than classical constructs like ‘reward.’ Interestingly, this prior over observations does not come into play when performing inference about hidden states (i.e., it is not part of the generative model in Equation (3)), but only during decision-making and action. Under active inference, actions are selected to minimise a quantity called the *expected free energy*, a quasi-value function that scores policies by their ability to bring expected observations in alignment with preferred observations, while also maximising information gain (see the Section 2.11 for more details). In the current model, we do not rely on this C vector to encode goals, but rather motivate action through a conditional action prior (see the section on the E vector below). For this reason, in our model the C is a flat distribution over observations and does not contribute to decision-making in this context.

#### 2.7.2. State Prior D

The prior over hidden states at the initial timestep is encoded by the so-called D vector, $P(s_{0}|D)$. The D vector encodes the agent’s beliefs about the initial state of the world, prior to having made any observations. In the context of the opinion formation generative model, it encodes baseline beliefs about which Idea is true, the meta-beliefs of the focal agent’s neighbours, as well as the initial tweet that the focal agent is making and the initial neighbour to whom the focal agent is attending.

#### 2.7.3. Empirical Prior over Hashtag Control State: $E^{T}$

We furnish the generative model with a special conditional prior over Hashtag control states $P(u_{0}^{T}|s^{\mathrm{Idea}})$, parameterised by a mapping denoted by $E^{T}$. This quasi-likelihood or link function renders the prior over Hashtag control states $u_{0}^{T}$ an *empirical prior*, because of an explicit dependence on $s_{t}^{\mathrm{Idea}}$. Under active inference, the final posterior over control states $Q(u_{t})$ becomes a Bayesian average of the ‘value’ of each control state, as determined by the (negative) expected free energy (see the corresponding Section 2.11 below), as well as the prior probability of each control state as encoded by $P(u_{0})$. In the current model, we make the prior over control states an *empirical prior* parameterised by a ‘link function’ denoted by the $E^{T}$ vector. This makes the prior over the Hashtag control state $u_{0}^{T}$ conditionally dependent on the $s^{\mathrm{Idea}}$ hidden state factor of the generative model. In practice, this implies that the prior over those control states corresponding to tweet actions $P(u_{0}^{T})$ depends on the posterior over $s_{t}^{\mathrm{Idea}}$, the hidden state corresponding to which Idea is true. This can be expressed as follows:

$$\begin{array}{cc} P(u_{0}^{T}|s_{t}^{\mathrm{Idea}}) & =\mathrm{Cat}(E^{T}) \end{array}$$

where the mapping encoded by the entries of $\mathrm{Cat}(E^{T})$ is an identity matrix that maps each value of $s^{\mathrm{Idea}}$ to a single Hashtag control state (value of $u_{0}^{T}$). At each timestep we approximate the prior at timestep *t* over $s^{\mathrm{Idea}}$ with the agent’s current posterior belief $Q(s_{t}^{\mathrm{Idea}})$. The following sections on belief updating explain how one optimises the variational posterior over hidden states $Q(s_{t})$ using observations. Once approximated in this way, we can re-express the empirical prior over Hashtag control states $P(u_{0}^{T})$ as:

$$\begin{array}{cc} P(u_{0}^{T}) & =E_{Q(s_{t})}\left[P\left(u_{0}^{T}|s_{t}^{\mathrm{Idea}}\right)\right] \end{array}$$

Agents are therefore more likely to take the action $u^{T}=\mathrm{Tweet}\mathrm{Hashtag}1$ if they believe more in **Idea 1** than in **Idea 2** (as reflected in the value of $Q(s_{t}^{\mathrm{Idea}})$), and likewise are more likely to take the action $u^{T}=\mathrm{Tweet}\mathrm{Hashtag}2$ if they believe more in **Idea 1** than in **Idea 2**. This empirical prior formulation thus renders the probability of taking a particular **Tweet Hashtag** action directly proportional to the agent’s belief in one of the two Ideas, as encoded in the variational posterior $Q(s_{t}^{\mathrm{Idea}})$.

#### 2.7.4. Prior over Neighbour Attendance Control State: $E^{\mathrm{Who}}$

In addition to the prior over Hashtag control states $P(u_{0}^{T})$, the generative model also contains a prior over the NeighbourAttendance control state $u_{0}^{\mathrm{Who}}$. We parameterise this prior over control states using a categorical distribution $E^{\mathrm{Who}}$, whose probability itself is given by a Dirichlet distribution with parameters ε:

$$\begin{array}{c} P\left(u_{0}^{\mathrm{Who}}|E^{\mathrm{Who}}\right)=E\left[Dir\left(\varepsilon \right)\right] \end{array}$$

The Dirichlet parameters ε, unlike the parameters of categorical distributions, are positive but not constrained to integrate to 1.0. As hyperparameters of a conjugate prior distribution, they are often analogised to ‘pseudo-counts’ that score the prior number of times a given action has been taken (in this case, sampling a particular neighbour via the control state $u_{0}^{\mathrm{Who}}$). For instance, if the ε vector for an agent with three neighbours is initialised to have the values (5,2,1), this means that the focal agent has a built-in propensity to take the action AttendNeighbour1 rather than the actions AttendNeighbour2 or AttendNeighbour3. Furthermore, in turn, taking the action AttendNeighbour2 is twice as probable as taking the action AttendNeighbour3. As we will see in the following sections, this ‘habit vector’ ε can be learned over time by optimising a variational beliefs over $E^{\mathrm{Who}}$, which involves incrementing a Dirichlet ε vector that parameterises the posterior $Q(E^{\mathrm{Who}})$.

### 2.8. Summary

This concludes the specification of a single agent’s generative model for opinion formation. Now that we have specified this generative model, we move on to define the family of the approximate posteriors (the agent’s beliefs) over hidden states and policies Q(s,π;ϕ) as well as the variational free energy. In conjunction with the generative model, these can be used to derive the update equations used to perform active inference.

### 2.9. Approximate Posteriors and Free Energy

Under active inference, both perception and decision-making are cast as approximate inference problems, wherein the variational free energy (or bound on surprise) is minimised in order to optimise beliefs about hidden states (perception) and beliefs about policies (decision-making/action). In order to derive the equations that perform this optimisation, we therefore have to define the variational free energy. This free energy, equivalent to the bound defined in Equation (2), requires both an approximate posterior and a generative model. We defined a POMDP generative model for our active inference agents in the previous section; the remaining step before writing out the free energy is then to define an approximate posterior distribution. For compatibility with the categorical prior and likelihood distributions of the generative model defined in Equation (3), we will also define the approximate posterior as categorical distributions. Additionally, we will invoke a particular factorisation of the approximate posterior, also known as a *mean-field approximation*, that allows us to factorise the approximate posterior over hidden states across timesteps. We define the approximate posterior over hidden states and policies as follows:

(16) $$\begin{array}{cc} Q(s_{\tau }|\pi ) & =Cat(s_{\pi \tau })\\ Q(\pi ) & =Cat(\pi )\\ Q(s_{1:T},\pi ) & =Q\left(\pi \right)\overset{T}{\prod_{\tau =1}}Q\left(s_{\tau }|\pi \right) \end{array}$$

where the notation P(x)=Cat(ϕ) denotes a categorical distribution over some random variable *x* with parameters ϕ. While this simplification assumes that posterior beliefs at subsequent timesteps are statistically independent, as we will see below, the Markovian temporal structure of the generative model means that, in practice, beliefs about hidden states at one timestep are contextualised by empirical priors from past timesteps (posterior beliefs from earlier timesteps).

The full free energy for the POMDP generative model and the approximate posterior specified in Equation (16) can be written as follows:

$$\begin{array}{cc} F_{1:T} & =E_{Q(s_{1:T},\pi )}\left[\ln Q\left(s_{1:T},\pi \right)-\ln P\left(o_{1:T},s_{1:T},\pi \right)\right] \end{array}$$

Equipped with the free energy, we can now derive update equations for hidden state estimation and policy inference that involve minimising $F_{1:T}$.

### 2.10. State Estimation

Under active inference, hidden state estimation is analogised to perception—this is achieved by optimising the variational posterior $Q(s_{1:T}|\pi )$ over hidden states, given policies. Because our approximate posterior and generative models are defined using categorical distributions, the problem of state estimation becomes minimising free energy gradients of the form $\frac{\partial F}{\partial s}$, where s are the parameters of the approximate posterior distribution over hidden states, Q(s)=Cat(s).

At each timestep, the agent can take advantage of the mean-field factorisation of the posterior and the Markovian structure of the generative model to update only its beliefs about the current state of the world: $Q(s_{t})$. The optimal posterior at timestep *t* is then found by finding the solution to $Q(s_{t})$ that minimises the timestep-specific free energy $F_{t}$:

(17) $$\begin{array}{cc} F_{t} & =E_{Q\left(s_{t}\right)Q\left(\pi \right)}\left[\ln Q\left(s_{t}\right)-\ln P\left(o_{t},s_{t}|s_{t-1},\pi \right)\right]\\ \Rightarrow \frac{\partial F_{t}}{\partial Q(s_{t})} & =0\Leftrightarrow Q^{*}\left(s_{t}\right)=\sigma \left(\ln P\left(o_{t}|s_{t}\right)+\ln\left(P\left(s_{t}|s_{t-1},u_{t-1}\right)P\left(s_{t-1}\right)\right)\right) \end{array}$$

This furnishes a simple belief update scheme for perception, where the optimal posterior $Q^{*}\left(s_{t}\right)$ is a Bayesian integration of a likelihood term $P(o_{t}|s_{t})$ and a prior term $P\left(s_{t}|s_{t-1},u_{t-1}\right)P\left(s_{t-1}\right)$.

Further details on the form of the approximate posterior and the derivation of the time-dependent free energy can be found in Appendix A.

### 2.11. Policy Inference

Under active inference, policies π are also a latent variable of the generative model and thus must be inferred. Accordingly, planning and action also emerge as results of (approximate) Bayesian inference, where now the inference is achieved by optimising a variational posterior over policies Q(π).

The optimal posterior that minimises the full variational free energy $F_{1:T}$ is found by taking the derivative of $F_{1:T}$ with respect to Q(π) and setting this gradient to 0, yielding the following free-energy-minimising solution for Q(π):

(18) $$\begin{array}{cc} Q^{*}\left(\pi \right) & =\underset{Q(\pi )}{\mathrm{argmin}}\;F=\sigma \left(\ln P\left(\pi \right)-F\left(\pi \right)\right) \end{array}$$

Therefore, in the same way that state estimation or optimisation of Q(s) in Equation (17) resembles a Bayesian average of a likelihood and a prior term, policy inference also becomes an average of the policy prior P(π) and the ‘evidence’ afforded to each policy, scored by F(π). See Appendix A for a more detailed derivation of the optimal policy posterior $Q^{*}\left(\pi \right)$.

The crucial component in understanding the behaviour of active inference agents lies in the specification of the policy prior, P(π). Under the standard construct of active inference (However, see alternative derivations as in [122,123]), the probability of a policy is defined a priori to be proportional to the negative *expected free energy* of that policy:

(19) $$\begin{array}{c} P(\pi )=\sigma (-G(\pi )) \end{array}$$

The expected free energy or EFE is denoted by G(π), and it measures the free energy *expected* under pursuit of a policy. This expected or predictive nature of the EFE is crucial: although the standard free energy is typically a direct function of observations (and functional of beliefs), when evaluating the consequences of a policy in the future, observations are not known—therefore, the expected free energy must deal with predicted observations or predictive densities over observations. As we will see below, this counterfactual nature of the expected free energy is what endows action selection with inherently both goal-directed and information-seeking components.

The expected free energy is defined mathematically as:

(20) $$\begin{array}{c} G\left(\pi \right)=D_{KL}[Q\left(s_{1:T},\pi \right)\left|\right|\tilde{P}\left(o_{1:T},s_{1:T},\pi \right)] \end{array}$$

where $\tilde{P}$ represents a generative model ‘biased’ towards the preferences of the agent. We can write this biased generative model at a single timestep as $\tilde{P}\left(o_{\tau },s_{\tau },\pi \right)=P\left(s_{\tau }|o_{\tau }\right)\tilde{P}\left(o_{\tau }\right)$, where $\tilde{P}\left(o_{\tau }\right)$ represents a ‘biased prior’ over observations. Given the factorisation of the approximate posterior Q(s,π) over time as defined in (16), the EFE for a single timestep can also be defined as follows:

(21) $$\begin{array}{cc} G{\left(\pi \right)}_{\tau } & =D_{KL}\left[Q\left(s_{\tau }|\pi \right)\|\tilde{P}\left(o_{\tau },s_{\tau }\right)\right]\\ \; & \approx -\underset{\mathrm{Epistemic}\;\mathrm{Value}}{\underset{︸}{E_{Q(o_{\tau }|\pi )}\left[D_{KL}\left[Q\left(s_{\tau }|o_{\tau },\pi \right)\|Q\left(s_{\tau }|\pi \right)\right]\right]}}-\underset{\mathrm{Utility}}{\underset{︸}{E_{Q(o_{\tau }|\pi )}\left[\ln\tilde{P}\left(o_{\tau }\right)\right]}} \end{array}$$

where the first term, the epistemic value, scores policies according to how much information observations $o_{\tau }$ expected under that policy provide about hidden states. This term is expressed here as the divergence between the states predicted under a policy, with and without conditioning on observations. The second term represents the degree to which expected outcomes under a policy will align with the biased prior over observations in the generative model. Since the prior over policies *minimises* expected free energy, policies with thus favoured states resolve uncertainty (maximise epistemic value) and satisfy prior preferences (maximise utility).

Having specified the prior over policies in terms of the (negative) expected free energy, we can now rewrite Equation (18) by expanding the prior in terms of G(π):

(22) $$\begin{array}{c} Q^{*}\left(\pi \right)=\sigma \left(-G\left(\pi \right)-F\left(\pi \right)\right) \end{array}$$

Additionally, in extensions introduced in [72], one has the option of augmenting the prior over policies with a ‘baseline policy’ or ‘habit vector’ $P(\pi_{0})$, also referred to as the E distribution. This means that the full expression for the optimal posterior can be written as (expanding lnP(π) as $\ln P(\pi_{0})-G$):

(23) $$\begin{array}{c} Q^{*}\left(\pi \right)=\sigma \left(-G\left(\pi \right)+\ln P\left(\pi_{0}\right)-F\left(\pi \right)\right) \end{array}$$

We introduce this ‘habit vector’ $P(\pi_{0})$ explicitly here, because it will be one of the parameters we explore in the multi-agent model. Note that in the Section 2.13 below, we reformulate the prior over policies in terms of two separate priors over *control states* in order to disentangle the prior over policies that include particular Hashtag control states $u^{T}$ from the prior over policies that are specific to neighbour-attendance control states $u^{\mathrm{Who}}$.

### 2.12. Action Selection

Action selection results from sampling from the marginal posterior over actions, or ‘control states’. The marginal posterior over actions can be computed by marginalising out the posterior probability of policies using the policy-to-control mapping $P(u_{t}|\pi )$:

(24) $$\begin{array}{cc} Q(u_{t}) & =\sum_{\pi }P\left(u_{t}|\pi \right)Q\left(\pi \right) \end{array}$$

This marginalisation is necessary because the mapping between policies and actions is not necessarily one-to-one: in the case of multi-timestep policies or multi-factor generative models, a particular control state $u_{t}$ might be entailed by more than one policy. Therefore, this marginalisation effectively computes the value of each action by summing together the posterior probabilities of all policies that include it. This entailment relation is encoded in the likelihood $P(u_{t}|\pi )$.

Once the posterior over control states $Q(u_{t})$ has been computed, an action *a* is simply sampled from this posterior marginal—this is then the action that the agent takes at timestep *t*:

(25) $$\begin{array}{c} a_{t}∼Q\left(u_{t}\right) \end{array}$$

### 2.13. Habit Learning

Under active inference, *learning* also emerges as a form of variational inference. However, this inference is not over hidden states, but rather over model parameters [72]. Such parameter inference is referred to as ‘learning’ because it is often assumed to occur on a fundamentally slower timescale than hidden state and policy inference. However, the update equations for model parameters follow the exact same principles as hidden state inference—namely, we optimise a variational posterior over model parameters Q(ϕ) by minimising the variational free energy F.

In the current model, we use ‘habit learning’ as originally described in [72] to model the development of so-called ‘epistemic habits,’ or the tendency for an originally epistemically motivated behaviour to become habitually driven, mimicking the transfer from model-based to model-free learning in the context of behavioural conditioning [75,76]. Technically, habit-learning reduces to updating a variational posterior over the categorical vector $E^{\mathrm{Who}}$, which parameterises the prior over the neighbour-attendance control state $u^{\mathrm{Who}}$.

Recall from the Section 2.7 that $E^{\mathrm{Who}}$ is a vector of categorical parameters whose prior probability is given as a Dirichlet distribution:

(26) $$\begin{array}{c} P\left(u_{0}^{\mathrm{Who}}|E^{\mathrm{Who}}\right)=E\left[Dir\left(\varepsilon \right)\right] \end{array}$$

The Dirichlet distribution is a conjugate prior for categorical distributions, meaning that the resulting posterior will also be Dirichlet distributed. Motivated by this conjugacy, we can define a variational posterior over the ‘habits’ $Q(E^{\mathrm{Who}})$ parameterised by variational Dirichlet parameters ε. One then simply augments the generative model from Equation (3) with the prior over the categorical $E^{\mathrm{Who}}$ parameters, which then allows one to define a new variational free energy, supplemented with the approximate posterior over $E^{\mathrm{Who}}$. Solving for the free-energy minimising solution with respect to the variational Dirichlet parameters ε leads to the following fixed-point solution for $Q(E^{\mathrm{Who}})$ [124]:

(27) $$\begin{array}{c} Q\left(E^{\mathrm{Who}}\right)=Dir\left(\varepsilon \right)\\ \varepsilon^{*}=\varepsilon +\eta \cdot Q\left(u_{t}^{\mathrm{Who}}\right) \end{array}$$

where η is a so-called ‘learning rate’ and $Q(u_{t}^{\mathrm{Who}})$ are current posterior beliefs about $u^{\mathrm{Who}}$ controls states. In other words, agents will update their posterior over actions or ‘habit vector’ according to how often they attend to a particular neighbour, as measured by the probability of each $u^{\mathrm{Who}}$ action. In the current work, we eschew the usual ‘separation of timescales’ assumption used in learning simulations (e.g., in [72,125]) and update the posterior habit vector ε at every timestep, i.e., after every action. This means that agents in this context simultaneously infer which neighbour to attend to, based on the prerogative to minimise expected free energy, while also incorporating a continuously learned ‘habit’ based on the frequencies with which they attend to different neighbours.

### 2.14. Multi-Agent Simulations

Now that we have introduced the generative model used by single agents and the ensuing inference, action, and learning rules that each agent will use to update its beliefs over time, we proceed to describe the multi-agent simulation itself.

A single multi-agent opinion dynamics simulation consists of a group of *N* active inference agents, where in the current work *N* ranged from 12–30 agents. Each agent is equipped with the single generative model of opinion formation, as described in the previous sections. All simulations described below were conducted using pymdp, a freely available Python package for performing active inference in discrete state spaces [126].

At each timestep, all agents simultaneously (1) update their beliefs as a function of observations and then (2) take an action (i.e., selecting which Hashtag to tweet and which neighbour to attend to). Crucially, each agent’s observations are a function of its own actions at the previous timestep, as well as the actions of a select set of neighbours at the previous timestep. Each agent has a fixed set of neighbours, where the particular neighbours are determined by a randomly chosen network topology. In the current study, we set the neighbour-to-neighbour connectivity for all simulations using Erdős-Rényi (ER) networks with some connection parameter *p*, meaning that agents are connected with fixed probability *p* [127]. For the current purposes, we make these networks undirected or symmetric, so that any agents that share an edge can both observe each other’s tweet actions and choose to read each other’s tweets. The components of each agent’s generative model (i.e., the number of observation modalities, number of hidden state factors) is a function of its local connectivity and the number of neighbours that it has. For example, a random agent in the network that was initialised to have three other neighbours will have three hidden state factors corresponding to the ‘meta-beliefs’ of these three neighbours: $s^{\mathrm{MB}1},s^{\mathrm{MB}2}$, and $s^{\mathrm{MB}3}$ as well as three observation modalities that it will use to read each of those neighbours’ tweets: $o^{\mathrm{NT}1},o^{\mathrm{NT}2}$, and $o^{\mathrm{NT}3}$. Each of those neighbouring active inference agents’ actions (which Hashtag they tweet) will thus feed into the focal agent’s various NeighbourTweet modalities at every timestep. Because edges are bidirectional, each of the neighbouring agents themselves will have a hidden state factor and observation modality, in their respective generative models, that represent the beliefs and **Tweet Hashtag X** actions of the focal agent.

In the results section to follow, we investigate the opinion dynamics under active inference by testing the hypotheses stated in the Section 1.6. We do this by systematically varying both the network connectivity *p* and the parameters of individual generative models, in an effort to investigate the extent to which ‘epistemic communities’ depend on both network properties and the cognitive features of individuals.

## 3. Model Parameterisation

### 3.1. Fixed Parameters

It is worth mentioning the vast parameter space one encounters when simulating multi-agent active inference models. In the current work, each active inference agent is equipped with an entire POMDP generative model that contains hundreds of individual parameters (consider, for example, all the categorical parameters that comprise the observation model $P(o_{\tau }|s_{\tau })$). Importantly, this parameter explosion is exacerbated in the multi-agent setting, since not only does the number of total parameters scale simply in the size of the network *N*, but connections between agents render this scaling supra-linear in *N*, since each agent is equipped with $K_{i}+2$ hidden state factors and observation modalities, where $K_{i}$ is the number of neighbours that agent *i* is connected to.

This means that the possible parameter space that one must explore in order to understand the behaviour of the model is combinatorially explosive. To enable transparency and efficient parameter exploration, we employ several simplifications and low-dimensional parameterisations of every agent’s generative model, which render the resulting space easier to explore.

First of all, we assume that every agent’s observation model relating the tweet content of others to their beliefs has the same basic form. Recall from Equation (7) the ‘Hashtag semantics’ matrix h that comprises the observation model for the observation of neighbour *k*’s tweet content: $P(o^{\mathrm{NT}k}|s)$, parameterised with a ‘Hashtag reliability’ parameter $p_{h}$. We fix this matrix to have the same parameter $p_{h}=0.73$ for all agents:

(28) $$\begin{array}{c} h=\left[\begin{array}{cc} 0.73 & 0.27\\ 0.27 & 0.73 \end{array}\right] \end{array}$$

The choice of $p_{h}=0.73$ was simply chosen since it is the setting of a two-element one-hot vector that is softmax-transformed using a precision parameter of 1.0. The choice of this particular parameter was not motivated by realism, or by construct- or ecological-validity. Instead, we chose it because it intuitively represents a “medium” level of precision, between the limits of precision →−∞ and precision →+∞, where the values of $p_{h}$ will converge to 0.5 and 1.0, respectively. When the precision is 1.0, the value of $p_{h}$ occupies a relative average of these extreme values at a value of 0.73. A focal agent believes that if it sees some neighbour *k* tweeting **Hashtag 1**, then the likelihood that neighbour *k* believes in **Idea 1** is 73%, and the likelihood that they believe in **Idea 2** is 27%. The relationship is inverted in case the focal agent sees neighbour *k* tweeting **Hashtag 2**. In the current study we assume that this basic Hashtag semantics matrix in Equation (28) is common to all agents, and for all neighbours (relative to some focal agent). This enables us to selectively explore the effect of *epistemic confirmation bias*, a single (scalar) precision γ that can be used to up- or down-weight columns of the Hashtag semantics matrix, according to whether a given neighbouring agent’s belief aligns with (the focal agent’s belief about) the environmental hidden state factor $s^{\mathrm{Idea}}$ (see the section on Neighbour Tweet Likelihood for a more detailed explanation).

Another restriction is in space of network architectures we explore; for the present study, we constrain the connectivity to be defined by random graphs (also known as Erdős–Rényi or ER networks) that are characterised by two parameters: the network size *N* and the connectivity *p*. We render the simulations computationally tractable by exploring small networks (in the range of N=12–30 agents) while systematically varying the connection probability *p*. We also assume that all agents’ transition models (those for both the environmental hidden state factor $s^{\mathrm{Idea}}$ and meta-belief factors $s^{\mathrm{MB}}$) are a scaled version of the 2×2 identity matrix $I_{2}$. This further enables their systematic exploration in terms of single scalar (the precision), rather than exploring all possible parameterisations of 2×2 transition matrices. In addition, while we systematically explore the inverse volatility parameter $\omega^{\mathrm{Soc}}$ and epistemic confirmation bias precision γ, we fix the value of $\omega^{\mathrm{Idea}}$ to be 9.0 for all simulations. We leave the full combinatorial exploration of all parameters, including $\omega^{\mathrm{Idea}}$, to future work.

Finally, while parametrically exploring the dependence of collective outcomes on individual parameters, we usually restricted parameter sweeps to vary at most two parameters at a time. We did this in order to simulate a sufficient number of trials for each condition while also investigating each parameter with as fine a resolution as possible. Under both these constraints, the computation time would explode when varying more than just two parameters simultaneously; we thus fix the values of the non-varied parameters to limit computational burden (e.g., fix $\omega^{\mathrm{Soc}}$ while varying γ and η). In practice, we clamped the value of the fixed parameters to ‘insensitive’ regions of parameter space where we know that the collective measure of interest (e.g., polarisation) did not depend on small changes in that parameter.

### 3.2. Parameters of Interest

In the following results section we describe four sets of parameters that we systematically varied to investigate their role in determining emergent phenomena in the multi-agent simulations. Below we briefly step through each parameter and rehearse its interpretation, and our motivation for investigating it.

#### 3.2.1. Epistemic Confirmation Bias

Recall from Section Neighbour Tweet Likelihood that *epistemic confirmation bias* or ECB is a precision parameter γ that selectively scales the Hashtag semantics matrix of the agent’s observation model, linking $s^{\mathrm{Idea}}$ and $s^{\mathrm{MB}k}$ to $o^{\mathrm{NT}k}$. The ECB precision γ scales the Hashtag semantics matrix in such a way that some focal agent *i* receives evidence for the $s^{\mathrm{Idea}}$ hidden state factor’s value (**Idea 1** vs. **Idea 2**) from the tweet output of some neighbour *k*, in proportion to how much neighbour *k* agrees with agent *i*.

This means that a focal agent with a higher γ believes that tweets more reliable if they come from neighbouring agents that are believed to share the opinion of the focal agent. The consequence of this is an ironically named ‘epistemic’ sort of confirmation bias, where agents believe that more reliable information about $s^{\mathrm{Idea}}$ comes from neighbours who are believed to be ‘like-minded’ to themselves. This can be revealed by recalling the expected free energy, the key determinant in action selection under active inference. As decomposed in Equation (21), this comprises an information gain term and a utility term. By means of the ECB parameter, the epistemic value term is preferentially higher for those actions that entail attending to a neighbour who the focal believes is like-minded. This can be analysed more quantitatively by inspecting the ‘negative ambiguity’ term of the epistemic value, H, which we show to be directly proportional to epistemic confirmation bias:

$$\begin{array}{cc} H=E_{Q(s^{\mathrm{Idea}},s^{\mathrm{MB}k})} & [\frac{1}{C}\left[p_{h}^{\gamma }\log p_{h}^{\gamma }+{\left(1-p_{h}\right)}^{\gamma }\log{\left(1-p_{h}\right)}^{\gamma }-2\log C\right]\\ \; & +\left[p_{h}\log p_{h}+\left(1-p_{h}\right)\log\left(1-p_{h}\right)\right]] \end{array}$$

See Appendix B for a complete derivation of the relationship between γ and epistemic value.

Given this relationship, we expect that higher epistemic confirmation bias will drive agents to preferentially attend to the actions of agents that share their beliefs. On a collective level, we hypothesise that ECB will increase the probability of both polarisation (two clusters of oppositely minded agents) and consensus (all agents have the same or similar beliefs about the **Idea**).

#### 3.2.2. Inverse Social Volatility

Recall the inverse temperature parameter introduced in Section 2.4, where we parameterised a focal agent’s beliefs about the stochasticity of the social dynamics using precision parameters $\omega^{\mathrm{Soc}}$ (following the notation used in [84]). The inverse social volatility scales the transition model that describes the dynamics of $s^{\mathrm{MB}k}$, such that a higher $\omega^{\mathrm{Soc}}$ induces an assumption of less stochasticity in the belief evolution of neighbours’ ‘meta-beliefs.’ This relationship also implies that the inverse social volatility is related to the epistemic value of actions that involve attending to particular neighbours. In particular, higher volatility (i.e., more entropy in the columns of the $B^{\mathrm{MB}k}$ matrices) leads to higher overall uncertainty in beliefs about hidden states. In other words, for lower values of $\omega^{\mathrm{Soc}}$ the uncertainty of the posterior marginal $Q(s^{\mathrm{MB}k})$ will accumulate faster, as long as the focal agent is not attending to neighbour *k*. Actions that entail attending to these unattended neighbours will therefore grow in epistemic value, the more time elapses during which those neighbours remain unattended. Importantly, the growth in epistemic value will scale inversely with $\omega^{\mathrm{Soc}}$ (see Appendix B for details). This means that the particular value of $\omega^{\mathrm{Soc}}$ sets an effective ‘refresh rate’ for how often a neighbour should be re-attended to, in order to resolve uncertainty about their beliefs.

Given this relationship, we hypothesise that high ‘meta-belief’ volatility (low $\omega^{\mathrm{Soc}}$) will lead agents to re-read their neighbours’ tweet content with a higher rate—whether or not they (believe they) agree with them—in order to resolve uncertainty about their beliefs. We expect that this continuous, epistemically driven ‘re-sampling’ will counteract the tendency of the group to polarise and thus favour collective agreement or consensus. An interesting question will be whether the inverse social volatility parameters directly ‘reverse’ the effect of γ, where the two jointly determine a collective trade-off between consensus and polarisation.

#### 3.2.3. Learning Rate

The learning rate η associated with updating the habit vector over neighbour-attendance control states $u^{\mathrm{Who}}$ represents the degree to which agents will preferentially sample those neighbours that they have attended to in the past. In the presence of a higher learning rate, the Dirichlet hyperparameters over the habit vector $E^{\mathrm{Who}}$ will be “bumped up" by a larger amount after choosing to attend to any particular agent, such that a focal agent will form preferences to attend to those agents whose Hashtags they habitually read. We expect therefore that a higher value of η will lead to increasingly preferential neighbour-attendance patterns among agents, and eventually to a change in the overall collective belief distribution of the group. Specifically, we hypothesise that ‘echo-chamber’-like dynamics will be exacerbated by a higher value of η, such that it will be harder to ‘escape’ from polarised dynamics in the presence of a large habit-learning rate η.

#### 3.2.4. Network Connectivity

In addition to individual generative model parameters like γ, $\omega^{\mathrm{Soc}}$, and η, we also quantitatively investigate whether and how the topology of agent-to-agent communication determines emergent behaviour. To quantitatively investigate this using a simple, one-dimensional parameterisation, we initialised the agent-to-agent communication network (i.e., which agents can read with other agents’ Hashtags) using a fixed random graph with connection probability *p*. For random graphs, *p* encodes the probability that any two agents have an edge between them. In the current context, an edge between any two agents determines whether they can view each other’s Hashtags, and thus form beliefs about one another’s beliefs). We hypothesise that denser communication topologies, represented by random graphs with increasing connection probability *p*, will obviate the risk of polarisation and lead to consensus with higher probability. In investigating this network effect, we also hope to reveal interactions between γ (which we hypothesise will *induce* polarisation) and connection probability *p*.

In the following sections, we describe the results of numerical experiments wherein we systematically vary the parameters discussed above, and reveal how they modulate the collective formation of ‘epistemic communities’ (e.g., echo-chambers, polarisation, and consensus).

## 4. Results

In the following sections we summarise the results of numerical experiments that validate the basic dynamics of the opinion formation generative model and then systematically investigate each of our three hypotheses. The results sections are organised as follows.

First, we demonstrate the basic dynamics of an active inference agent engaged in opinion formation. Over time, we show how a single focal agent updates its beliefs about the world in the face of conflicting Hashtag observations from two neighbours. In this process, the agent simultaneously forms beliefs about the abstract, environmental hidden state (**Idea 1** vs. **Idea 2**) as well as beliefs about the meta-beliefs of two neighbouring agents to whose Hashtags it is exposed. We examine the dependence of a single agent’s belief-updating dynamics on different settings of the epistemic confirmation bias γ and the inverse social volatility $\omega^{\mathrm{Soc}}$ under a fixed value of $\omega^{\mathrm{Idea}}=9.0$.

Next, we demonstrate the emergent formation of epistemic communities and the diverse dynamics that can be observed under the current active inference model. These are meant as proof-of-principle validation of the opinion dynamics model and the rich sorts of collective behaviours it can give rise to.

Finally, in order to test the three hypotheses that frame our study of epistemic communities under active inference, we systematic vary parameters such as γ, $\omega^{\mathrm{Soc}}$, η, and *p* to investigate how they determine collective dynamics. In these collective dynamics experiments, we link groups of active inference agents together and simulate their multi-agent dynamics for up to T=100 timesteps. We then study collective outcomes by averaging the results of hundreds of independent realisations.

When systematically varying parameter configurations, we define a single condition as a combination of the parameters of interest. This includes the network connectivity *p* and a vector of generative model parameters, e.g., $\gamma =3.5,\omega^{\mathrm{Soc}}=0.5,\eta =1.5$. For each condition, we ran 100 independent multi-agent simulations with a network size N=15 agents. We chose relatively small networks in order to limit the computational burden of each simulation.

### 4.1. Opinion Formation in a Single Agent

**Figure 2:**
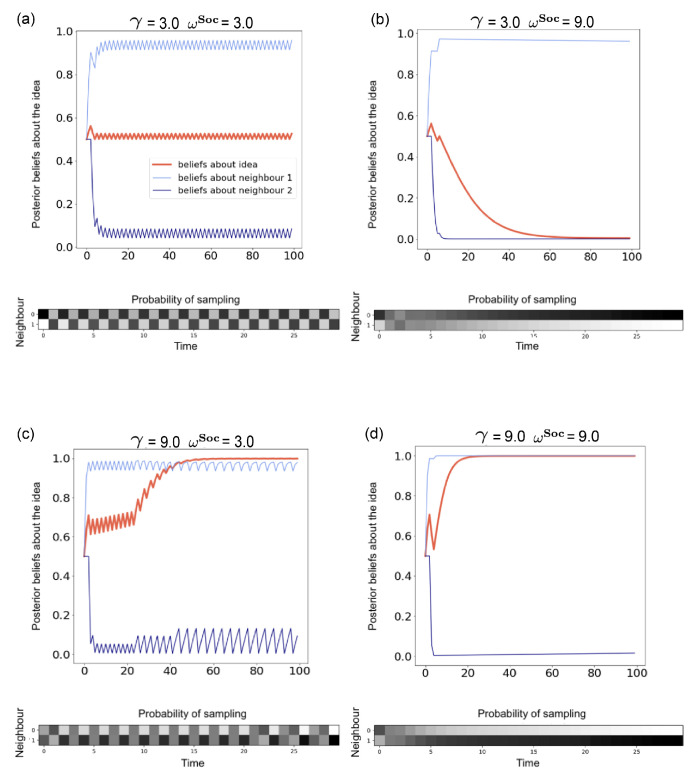
Belief dynamics and actions of a single agent in response to a sequence of Hashtag observations from two fictive neighbours. Shown are the history of Bernoulli parameters defining three marginal posterior beliefs of the focal agent: the belief about the truth value of **Idea 1** ($Q(s_{t}^{\mathrm{Idea}}=\mathrm{Idea}1)$, in red), and its beliefs about the beliefs of its two neighbours regarding **Idea 1** ($Q(s_{t}^{\mathrm{MB}1}=\mathrm{Idea}1)$ and $Q(s_{t}^{\mathrm{MB}2}=\mathrm{Idea}1)$, shown in two shades of blue). Through its generative model, the focal agent believes that its Hashtag observations are caused by two neighbour ‘meta-belief’ states. The focal agent is exposed to a sequence of Hashtag observations for 100 timesteps, where in case of attending to the first neighbour ($u_{t}^{\mathrm{Who}}=0$), the agent receives observation $o_{t}^{\mathrm{NT}1}=$**Hashtag 1**, $o_{t}^{\mathrm{NT}2}=$**Null**, and in case of sampling the other neighbour ($u_{t}^{\mathrm{Who}}=0$), the agent receives observation $o_{t}^{\mathrm{NT}1}=$**Null**, $o_{t}^{\mathrm{NT}2}=$**Hashtag 2**. Due to the ‘Hashtag semantics’ matrix in its generative model, these two Hashtags, respectively, lend evidence for the two levels of $s^{\mathrm{Idea}}$. At each timestep the focal agent performs inferences with respect to hidden states $Q(s_{t})$ as well as policies (control states) $Q(u_{t}$), and then samples a **Neighbour Attendance** action from the posterior over control states $Q(u^{\mathrm{Who}}=0,u^{\mathrm{Who}}=1)$. Below each subplot is a heatmap showing the temporal evolution of the probability of sampling Neighbour 1 vs. Neighbour 2 over time. Subfigure (**a**) shows an agent with low γ (3.0) and low $\omega^{\mathrm{Soc}}$ (3.0). The agent’s beliefs about both of their neighbors does not lead it to converge on an idea being true or not. Subfigure (**b**) shows an agent shows an agent with low γ (3.0) and high $\omega^{\mathrm{Soc}}$ (9.0). The agent will be more certain about the beliefs of their neighbors, attend less often to their neighbors, quickly converging to neighbour 2. Subfigure (**c**) shows an agent shows an agent with high γ (9.0) and low $\omega^{\mathrm{Soc}}$ (3.0). This agent believes in high volatility and will be driven to continue sampling their neighbors, which will lead them to take longer to converge towards an idea. However, given their γ, the agent does converge towards the first sampled idea. Subfigure (**d**) shows an agent shows an agent with high γ (9.0) and high $\omega^{\mathrm{Soc}}$ (9.0). This agent believes in low volatility and will be driven to sample the same neighbor very quickly, which will lead them to converge towards an idea quickly.

Figure 2 visualises opinion formation in a single active inference agent, and sheds light on the relationship between $\omega^{\mathrm{Soc}}$ and γ in determining the rate of belief updating and action selection. We investigate this using a simplified three-agent set-up, where one focal agent is exposed to a sequence of conflicting information from two neighbours. At each timestep, the focal agent chooses to read a Hashtag from one of its two neighbours, and the two neighbours are not actually active inference agents, but are simply sources of a sequence of discrete Hashtag observations (**Hashtag 1** issue from Neighbour 1, **Hashtag 2** issue from Neighbour 2). We can see anecdotally how belief updating and sampling behaviours are bidirectionally modulated by different combinations of $\omega^{\mathrm{Soc}}$ and γ. In general, Figure 2 shows that beliefs in more meta-belief volatility (lower $\omega^{\mathrm{Soc}}$) lead to higher posterior uncertainty about the $s^{\mathrm{Idea}}$ hidden state, as is shown by the red lines in subplots (a) and (c). Higher epistemic confirmation bias γ, on the other hand, induces a positive feedback effect, wherein the focal agent comes to agree with one of its two neighbours with high certainty, most likely whichever neighbour it happens to attend to at the first timestep.

With high enough γ or high enough $\omega^{\mathrm{Soc}}$, the focal agent’s beliefs, faced with these two conflicting sources of information, converge to one Idea. This choice is consistently reinforced by the focal agent’s continuing to sample the agent it agrees with (lower insets in each subplot of Figure 2). There is also an interesting interaction between γ and $\omega^{\mathrm{Soc}}$, such that $\omega^{\mathrm{Soc}}$ drives down posterior uncertainty in the focal agent’s beliefs about its neighbour $Q(s^{\mathrm{MB}k})$. This in turn decreases the information gain term in the expected free energy, such that the agent has stronger prior beliefs about its neighbour’s beliefs and there is less information gain afforded to attending to that neighbour. On the other hand, higher γ drives up epistemic value, even in the face of precise beliefs about the neighbour’s belief state, making the agent expect to artificially resolve more uncertainty from its observations.

It is clear that for configurations with high inverse social volatility, as the focal agent’s beliefs converge toward the beliefs of Neighbour 1, it also begins to attend to Neighbour 1 more often than Neighbour 2 (subplot (d)). However, with low inverse social volatility, the focal agent is driven to periodically attend to both neighbours, due to the increasing epistemic value associated with neighbours that are unattended to. Interestingly, when $\omega^{\mathrm{Soc}}$ is low and γ is high (Figure 2c), the focal agent continues to periodically re-attend to the neighbour it disagrees with, due to increasing uncertainty about that neighbour’s belief, induced by high volatility associated with it. Note, however, that the total probability of attending to the like-minded neighbour is still higher due to the presence of high epistemic confirmation bias. In the presence of both low epistemic confirmation bias and low inverse social volatility, posterior uncertainty is high all-around and the focal agent is ‘ambivalent’ between both **Idea 1** and **Idea 2**. Nonetheless, the focal agent succeeds in inferring the belief-states of its two neighbours as it repeatedly alternates between sampling them.

### 4.2. Epistemic Community Dynamics

**Figure 3:**
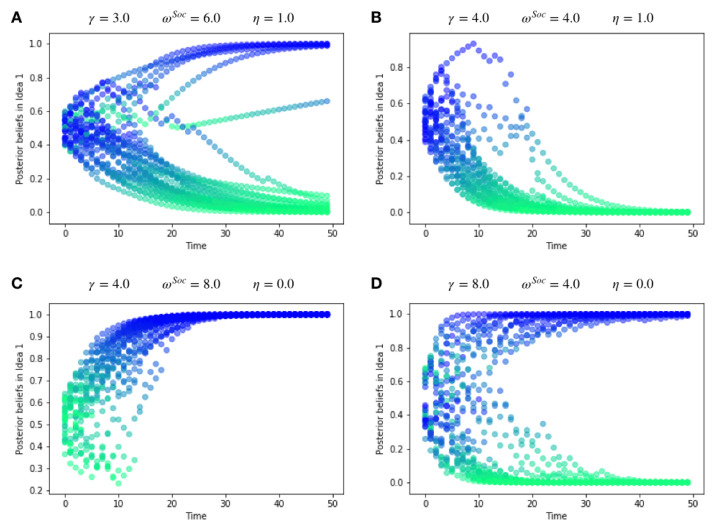
Collective belief dynamics of multi-agent active inference simulations under different generative model parameterisations. Above each panel are listed the parameter values of γ, $\omega^{\mathrm{Soc}}$, and η used in the simulation. Other parameters were fixed with T=50 timesteps, N=30, network connectivity p=0.2, and inverse environmental volatility $\omega^{\mathrm{Idea}}=9.0$. At the beginning of each simulation, every agent’s beliefs about **Idea 1** were sampled from a uniform distribution over the interval $Q\left(s^{\mathrm{Idea}}=\mathrm{Idea}1\right)\in \left(0.4,0.6\right)$. Each panel displays the evolving beliefs of all agents about **Idea 1** (the Bernoulli parameter of each agent’s respective posterior over $s^{\mathrm{Idea}}$), with proximity of the belief to 1.0 indicated by colouring along the green-to-blue spectrum (blue beliefs indicate $Q(s^{\mathrm{Idea}}=\mathrm{Idea}1)>0.5$). Panels (**A**,**D**) demonstrate polarisation, where two subsets of agents end up believing in two different levels of the **Idea** hidden state with high certainty. Panels (**B**,**C**) on the other hand show examples of consensus, where the whole network converges to the same opinion by the end of the simulation.

Figure 3 shows examples of the collective opinion dynamics (i.e., ‘epistemic communities’) that emerge when simulating networks of active inference. Unlike in Figure 2, in these simulations the observations for every agent are generated by the actions of other active inference agents, who are all collectively reading the Hashtag actions of other agents while and generating their own. We include this to showcase the rich phenomenology displayed by collectives of active inference agents, validating our model alongside known opinion dynamics models that can capture phenomena like consensus and polarisation. In the following sections we investigate the dependence of these dynamics on the parameters of generative models and network density quantitatively.

### 4.3. The Dependence of Epistemic Communities on γ and *p*

We first investigated Hypothesis 1, or how epistemic confirmation bias γ and network connectivity *p* determine the collective formation of epistemic communities. We systematically varied both epistemic confirmation bias (15 values of γ tiling the range (3,9)) and network connectivity (15 values of *p* tiling the range (0.2,0.8)) in networks of N=15 agents, and simulated S=100 independent realisations of each condition for T=100 timesteps. Other parameters were fixed to constant values ($\omega^{\mathrm{Soc}}=0.6$, $\omega^{\mathrm{Idea}}=9.0$, η=0.0). Note that here, habit-learning was intentionally disabled (η=0.0) to selectively investigate the effect of γ while excluding the effect of habit learning on epistemic community formation. Within each parameter configuration, every independent realisation and every agent had the same average value of epistemic confirmation bias γ, but for each agent, we sampled a vector of epistemic confirmation bias values from a normal distribution centred at the parameter setting with a variance of 0.1. Note that there are *k* different ECB parameters per agent because each agent has a collection of $A^{\mathrm{NT}k}$ arrays, each corresponding to the observation model from a particular neighbour. Each of these *k* likelihood arrays is parameterised by a single γ. By sampling γ across $A^{\mathrm{NT}k}$ arrays within each agent’s generative model, we implicitly gave each agent a particular bias to believe that certain neighbours were more ‘reliable’ than others—some neighbours contribute more or less to the focal agent’s confirmation bias tendency. Note that this same sort of across-neighbour sampling was performed for the inverse social volatility $\omega^{\mathrm{Soc}}=0.6$—in other words, 0.6 served as the mean of a normal distribution, from which each agent’s vector of neighbour-specific $\omega^{\mathrm{Soc}}$ parameters was sampled, one for each $B^{\mathrm{MB}k}$.

The aim was to investigate how higher epistemic confirmation bias, particularly in a sparse network, might drive the emergence of epistemic communities through the formation of belief clusters that are both dense and far apart in ‘belief-space.’ In general, it is known in the literature that clusters are more easily formed in sparsely connected networks, but less so in densely connected networks where all agents communicate with each other [94]. Therefore, one interesting hypothesis for this experiment was that increasing the value of γ could achieve the opposite effect: namely, a high degree of polarisation or belief-clustering behaviour in a densely connected network.

To assess the emergence of epistemic communities or clusters of like-minded individuals, we defined the polarisation index ρ, which measures the degree of ‘epistemic spread’ in a system. It is defined as the difference between the highest and the lowest values of the Bernoulli parameter defining $Q(s^{\mathrm{Idea}}=\mathrm{Idea}1)$ across all agents at the final timestep of the simulation (where the choice of one ‘side’ of the belief $Q(s^{\mathrm{Idea}}=\mathrm{Idea}1)$ is arbitrary). This final difference is then averaged across *S* independent realisations or trials to give the average value 〈ρ〉 for a particular condition. This is directly proportional to the ratio of the number of trials in any configuration in which the simulation ends with two opposing clusters, as opposed to consensus, where consensus is defined at the final timestep when all agents’ posterior beliefs about $s^{\mathrm{Idea}}$ are on the same side of 0.5.

(29) $$\begin{array}{cc} \rho_{s} & =\max_{i}\left[Q\left(s_{i}^{\mathrm{Idea}}=\mathrm{Idea}1\right)-\min_{i}Q\left(s_{i}^{\mathrm{Idea}}=\mathrm{Idea}1\right)\right]{|}_{T=100}\in \left[0,1\right]\\ \langle \rho \rangle & =\frac{1}{S}\sum_{s=1}^{S}\rho_{s} \end{array}$$

where *S* indicates the number of total trials (here, S=100).

A high value of ρ (close to 1) indicates more spread-out beliefs and implies clustering, i.e., echo-chamber formation, whereas a low ρ implies that the network of agents have similar beliefs about $s^{\mathrm{Idea}}$ (i.e., consensus).

**Figure 4:**
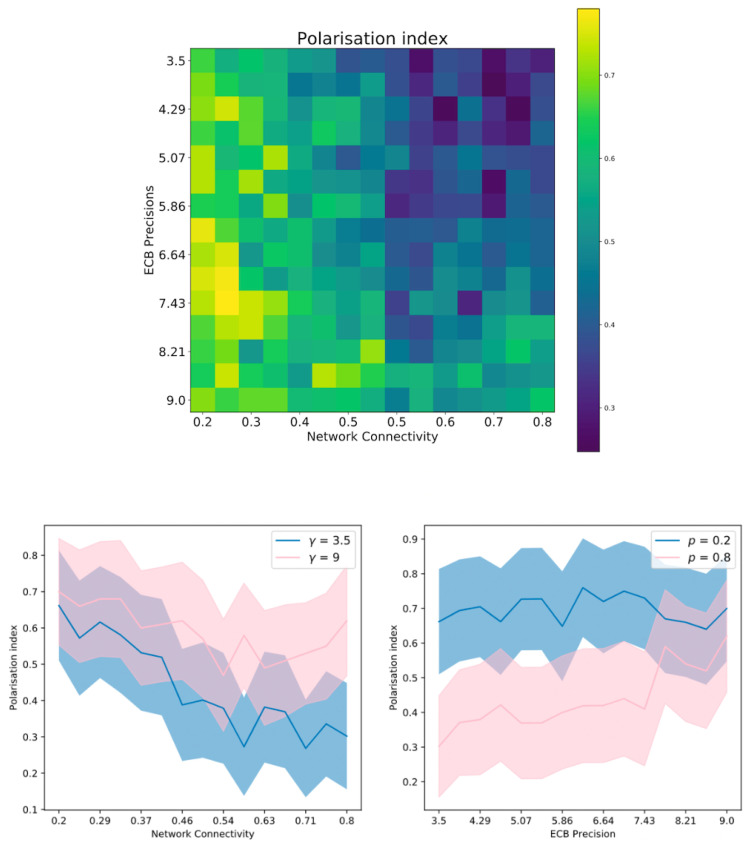
The interaction between epistemic confirmation bias and network connectivity in determining collective outcomes. (**Top**) a heatmap of the mean polarisation index across S=100 independent realisations of the multi-agent opinion dynamics simulations, for unique combinations of network connectivity *p* and epistemic confirmation bias precision γ. (**Bottom**) selected line plots show extreme settings of *p* (p=0.2 and p=0.8) and γ (γ=3.5 and γ=9.0). Shaded areas around each line represent the standard deviation of the polarisation index across independent realisations.

Figure 4 shows the effects of varying γ and *p* on polarisation as measured by 〈ρ〉. It is clear from the first column of the heatmap that highly spread out beliefs can occur at all values of the epistemic confirmation bias in the presence of sparse connectivity. Denser networks in general reduce the risk of polarisation, as seen by a drop-off in 〈ρ〉 as *p* increases. However, epistemic confirmation bias can ‘counteract’ this effect to some extent by marginally bumping up the risk of polarisation, even in the presence of denser networks (high γ and high *p*). The lower subplots of Figure 4 demonstrate this counteractive effect, where even at high connectivities (e.g., p=0.8) the epistemic confirmation bias can lead to the majority of trials resulting in polarised dynamics.

Why, one might wonder, does polarisation still occur with some probability even when γ is small? When network connections are sparse, polarisation can still occur by virtue of the agents lacking access to a variety of neighbours—this forces them to attend to one of a limited set of neighbours that they start out connected to. Since all agents are initialised with flat prior beliefs about $s^{\mathrm{Idea}}$, this leads to the formation of two clusters, since there is nothing correlating the beliefs of agents who are disconnected. Because there are two beliefs (**Idea 1** and **Idea 2**), this means that on average this fragmentation leads to distinct sub-clusters of connected agents that will believe in one of the two Ideas with approximately 50% probability.

As γ increases, even in the presence of increasing connectivity, agents are driven by epistemic value to preferentially attend to the neighbours that (they believe) share their beliefs. This accounts for the slower decrease in polarisation with increasing connectivity *p* at higher levels of γ shown in Figure 4. This can be compared to the faster decrease in polarisation induced by *p* when γ is low (compare the first few rows of the heatmap in Figure 4 to the last few rows).

However, network connectivity seems to be a stronger effect than γ in enforcing consensus or at least the lack of polarisation. This is because the exploration entailed by γ encourages agents to attend to a larger group of neighbours, leading to a higher average spread of beliefs and the ability for agents to serendipitously encounter other agents they agree with. However, because of the density of the network, it is much more difficult for agents to become polarised as they will more frequently be exposed to new information, despite their propensity towards confirmation bias.

### 4.4. Effect of Inverse Social Volatility on Neighbour Attendance and Polarisation

Next, we explored Hypothesis 2, modelling behaviour under different values of inverse social volatility $\omega^{\mathrm{Soc}}$ to see how it would interact with γ. We swept over γ (15 values tiling the range (3,9)) and $\omega^{\mathrm{Soc}}$ (15 values tiling the range (0.05,1.0)) in networks of N=15 agents with p=0.4 connection probability. As explained before, each agent’s generative model was parameterised by a vector of *k* distinct γ and $\omega^{\mathrm{Soc}}$ parameters, which were sampled from a normal distribution centred around the parameter value characterising the condition. In this case, each sampled value parameterised the different neighbour-specific observation ($A^{\mathrm{NT}k}$) and transition models ($B^{\mathrm{MB}k}$) for a particular focal agent.

To assess the extent to which social attendance changes as a function of γ and $\omega^{\mathrm{Soc}}$, we defined the re-attendance rate *r*. It scores the maximum number of times an agent samples the same neighbour throughout a parameter configuration, averaged over trials.

$$\begin{array}{cc} r_{s} & =\max_{i}\sum_{t}1_{i}\left(u_{t}^{\mathrm{Who}}\right)\\ \langle r\rangle & =\frac{1}{S}\sum_{s=1}^{S}r_{s} \end{array}$$

where 1 is the indicator function.

**Figure 5:**
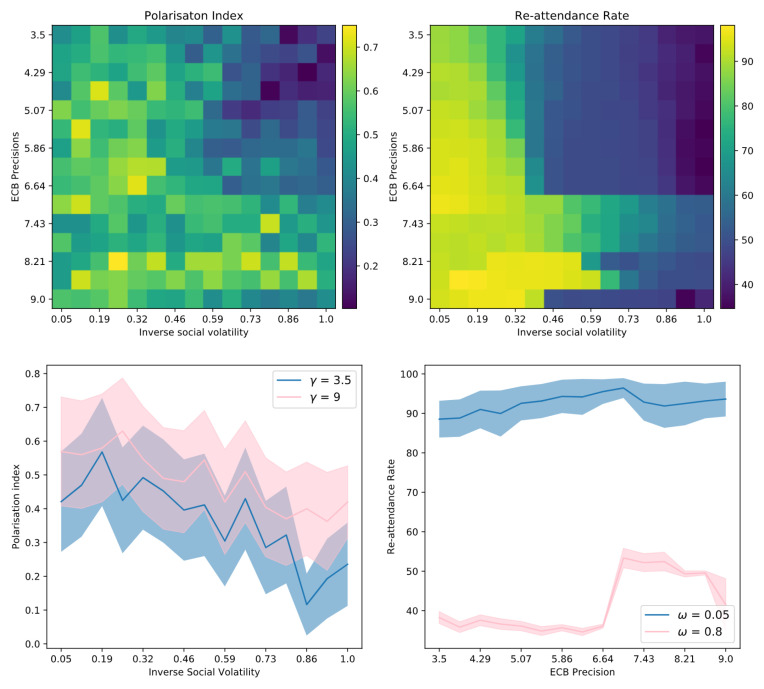
(**Above left**) a heatmap of the polarisation index for all 225 combinations of inverse belief volatility and epistemic confirmation bias precision. (**Above right**) a heatmap of the re-attendance rate for all 225 combinations of inverse belief volatility and epistemic confirmation bias precision. (**Below left**) a line plot of the most extreme rows of the polarisation heatmap. (**Below right**) a line plot of the most extreme columns of the re-attendance rate heatmap.

We measured the re-attendance rate and polarisation index for each configuration, averaged across trials. Figure 5 portrays a complex picture on the relationship between γ and $\omega^{\mathrm{Soc}}$. In the case of high volatility over meta-beliefs (low inverse social volatility), agents are driven to periodically re-attend to neighbours in order to resolve growing uncertainty about their beliefs. This is indicated by a higher average re-attendance rate 〈r〉 (top right heatmap). Interestingly, there is an interaction between re-attendance rate and epistemic confirmation bias, such that in the presence of low volatility (high inverse social volatility) *and* low epistemic confirmation bias, the re-attendance rate is minimised. We speculate that a low value of ECB (γ=3.5) makes the epistemic value of attending to every neighbour equally high purely a function of $\omega^{\mathrm{Soc}}$. In this case, agents will continually revisit neighbours sequentially, with the attendance-preference for any given neighbour solely dependent on the time elapsed since the last time they were attended to. In the absence of confirmation bias (which normally accelerates the focal agent’s beliefs not only about $s^{\mathrm{Idea}}$ but also about $s^{\mathrm{MB}k}$ (cf. Figure 2), this means that uncertainty about neighbours’ beliefs will on average be higher. This will lead to diverse social attendance patterns, such that agents will prefer to constantly sample new neighbours, with no particular neighbour excluded from this uncertainty-driven re-sampling. There seems to be a stark threshold around $\omega^{\mathrm{Soc}}=0.4$ above which the re-attendance rate drops off quite rapidly, as long as γ<6.64. This threshold probably represents the level at which the epistemic value induced by posterior uncertainty (which is a function of $\omega^{\mathrm{Soc}}$) surpasses the contribution to epistemic value induced by γ. For higher values of γ (>6.64), the drop off in re-attendance rate with increasing $\omega^{\mathrm{Soc}}$ is countered due to the increased contribution of γ to the epistemic value of repeatedly sampling neighbours who are believed to be like-minded. Finally, at high enough values of γ (≈9.0), the drop-off of re-attendance rate with increasing $\omega^{\mathrm{Soc}}$ is re-established. This is likely due to the ‘self-inhibiting’ effect of increasing γ on epistemic value. When γ is high enough, agents come to form very precise beliefs about their neighbours’ beliefs (the entropy of $Q(s^{\mathrm{MB}k})$ decreases), which in turn decreases the resolvable uncertainty about those neighbours’ beliefs. We refer the reader to Appendix B for a more quantitative exploration of this effect that exposes the epistemic value in terms of an entropy term and a negative ambiguity term.

In terms of polarisation, it seems that for γ<6.64, high volatility (low $\omega^{\mathrm{Soc}}$) encourages polarisation more than low volatility, since agents are driven to re-sample their neighbours (with whom they are likely to agree, due to epistemic confirmation bias), and will end up forming distinct belief clusters. However, for γ>6.64 the effect of $\omega^{\mathrm{Soc}}$ seems to disappear and we see high polarisation for all values of $\omega^{\mathrm{Soc}}$. We originally hypothesised that if agents are uncertain about the beliefs of their neighbours (low $\omega^{\mathrm{Soc}}$), it will become *more* difficult to induce polarisation and purposefully sample those who are thought to agree, due to the competing epistemic value induced by high posterior uncertainty about one’s neighbours’ beliefs, regardless of whether (a focal agent believes) those neighbours to be like-minded. However there is no clear difference between polarisation levels when γ is high. Nevertheless it is clear that higher γ induces polarisation for non-volatile networks. A more robust effect is how social volatility induces the tendency to re-attend to neighbours (cf. right panels).

### 4.5. Habit Formation and Network Initialisation

For the final experiment, we explored Hypothesis 3, regarding the polarisation of networks via habit formation. We swept over γ (15 values tiling the range (3,9)) and η (15 values tiling the range (0.0,0.9)) in networks of N=15 agents with p=0.4 connection probability, where $\omega^{\mathrm{Soc}}=0.6$ and as before $\omega^{\mathrm{Idea}}=9.0$. Here, γ was again normally distributed with a fixed mean (which varied by condition) and variance 0.1 across the *k* neighbours of each focal agent, but the learning rate η was fixed to the condition-dependent value across all trials and agents.

The learning rate η incentivises agents to re-attend to the same neighbour by forming a habit, which competes with the epistemic value of attending to a new neighbour with unknown beliefs. This experiment tested the hypothesis that a higher learning rate, i.e., stronger habit-formation, will increase polarisation.

**Figure 6:**
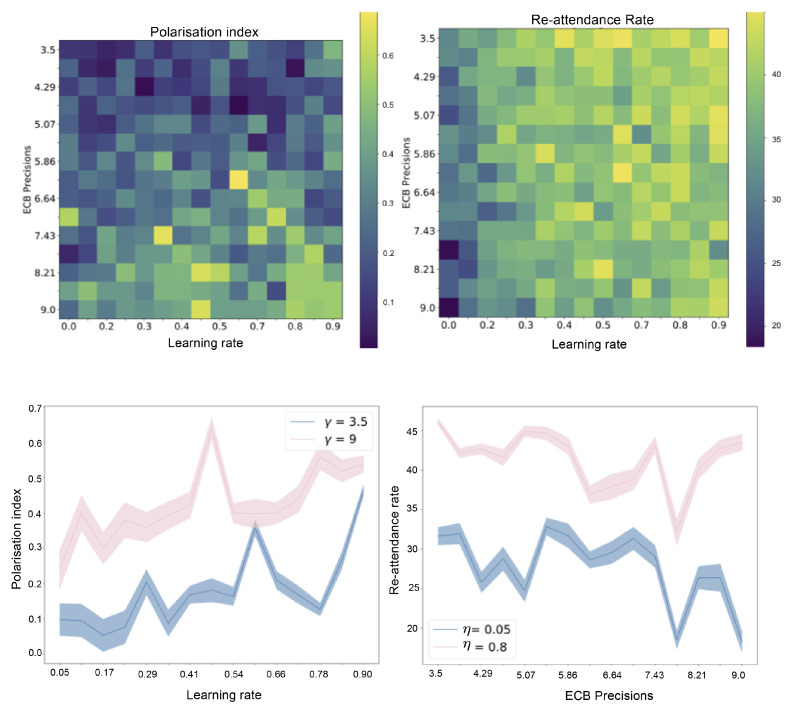
(**Above left**) a heatmap of the polarisation index for all 225 combinations of learning rate and epistemic confirmation bias precision. (**Above right**) a heatmap of the re-attendance rate for all 225 combinations of learning rate and epistemic confirmation bias precision. The parameters represent the centres of the normal distribution sampled from across trials for each configuration. (**Below left**) the most extreme row of the polarisation index heatmap. (**Below right**) the most extreme column of the re-attendance rate heatmap.

**Figure A1:**
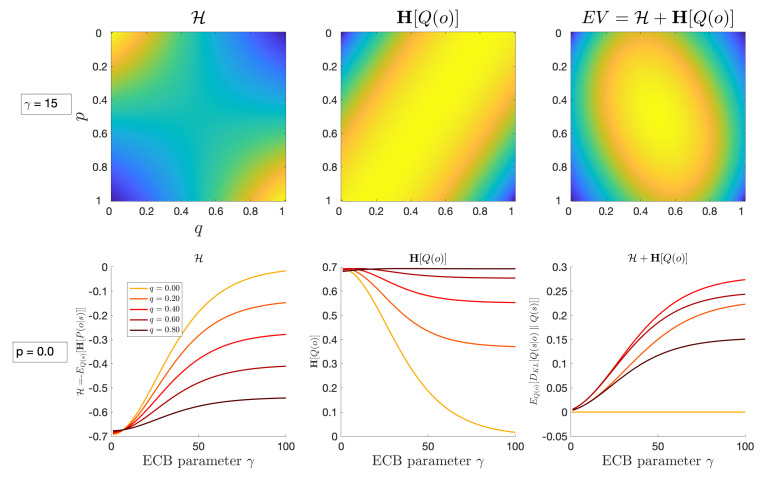
Exploration of the relationship between ‘agreement’ between an agent and one of its neighbours, the epistemic confirmation bias parameter γ, and the epistemic value of reading that neighbour’s tweet content. Here, the two marginal posteriors $Q(s^{\mathrm{Idea}})$ and $Q(s^{\mathrm{MB}k})$ are expressed as two Bernoulli distributions with respective parameters *p* and *q*, where ‘agreement’ is the case when p=q and hence (1−p)=(1−q). The top row shows heatmaps of the negative ambiguity H, entropy H[Q(o)], and the full epistemic value EV=H+H[Q(o)] for a fixed value of γ=15.0, under all possible values of *p* and *q*. The ‘epistemic confirmation bias‘ effect is seen in the negative ambiguity surface H (upper left plot), which is maximised when posterior beliefs about the validity of **Idea 1**, measured by *p*, are aligned with posterior beliefs about a neighbour’s meta-belief about **Idea 1**, *q*. The bottom row of plots shows a complementary perspective, demonstrating the effect of increasing γ on the epistemic value and its components, for different settings of *q* when p=0.0. The subplot on furthest to the right of the bottom row shows that increasing γ increases epistemic value most when *q* is on the same side of 0.5 as *p* (q=0.2,q=0.4), and the effect of γ on epistemic value deceases once *q* passes 0.5. Note that the epistemic value is 0 when p=q=0, because although the negative ambiguity is maximised in this case, it is counteracted by the entropy term which is 0 since both posteriors are certain.

Figure 6 demonstrates how learning rate η and epistemic confirmation bias γ interact to influence outcomes at the collective level. Indeed, a higher learning rate induces more polarisation, implying the formation of more ‘stubborn’ epistemic communities in the network. This effect appears at both low and high levels of epistemic confirmation bias, with on average a higher 〈ρ〉 observed with increasing learning rate, even at low levels of γ. However, it seems the effect is most pronounced at the highest levels of γ and η. Examining the average re-attendance 〈r〉 (right column of Figure 6) reveals a clear effect of η on neighbour re-attendance, with the rate seemingly maximised when the learning rate surpasses a value of η≈0.3. Interestingly, the effect of ECB on re-attendance is not very strong here, although it seems to have a mild negative effect. Namely, as ECB increases, the re-attendance rate tends to decrease. One counterintuitive explanation for this effect (which is similar to the effect observed in Figure 5) is the general increase in the epistemic value of attending to neighbours with unknown beliefs that is caused by increasing γ. Although by design γ is intended to ‘boost’ the epistemic value of only those actions that involve attending to neighbours that the focal agent believes it agrees with, there is still an overall ‘exploration bonus’ that scales with γ, *even for actions that entail attending to neighbours with whom the focal agent disagrees*. This is because in addition to the ambiguity term of the epistemic value, which captures the ‘confirmation bias’ effect encoded by γ, there is also a maximum-entropy component $H[Q\left(o_{\tau }|\pi \right)]$ (see Appendix B for details). This term is maximised when the posterior uncertainty over meta-beliefs $Q(s^{\mathrm{MB}})$ is high (maximal when $\left(s^{\mathrm{MB}}\right)=\left(0.5,0.5\right)$). Therefore, although ECB ‘bends’ the epistemic value landscape towards sampling like-minded neighbours (see Figure A1 in Appendix B for a visualisation of this effect), when compared to neighbours with differing beliefs, the inherently uncertainty-resolving nature of the epistemic value as a whole means that higher γ still increases the value of actions that involve attending to *any* neighbours whose beliefs the focal agent is uncertain about. This may in fact counteract the polarising effects we originally intended to capture by including the ECB parameter. This across-the-board ‘exploration bonus’ conferred by ECB may explain the mild effect we observe here, where increasing γ ends up decreasing average re-attendance 〈r〉. This may indeed explain the decrease observed in Figure 5 and Figure 6.

## 5. Discussion

In this paper, we focused on the way communities form around shared beliefs about abstract entities or meanings, symbolised by an abstract discrete hidden state: an ‘Idea’. Shared belief around a particular ‘Idea’ emerges through coordination, which itself is individually driven by the desire to form accurate (Bayesian beliefs) about the world and the beliefs of one’s community. In particular, we modelled confirmation bias as an ‘epistemic’ phenomenon wherein agents have a biased belief that agents with whom they believe they agree are more likely to provide uncertainty-resolving (information-availing) data—hence the proposed terminology of *epistemic confirmation bias*.

Twitter provides fertile ground for the academic study of the spread of ideas. The platform is extremely popular, easy to access, and has an API that enables researchers to collect and analyse data. It has also been one of the major vectors for misinformation, leading to large-scale events, like the tensions around the 2016 election results [128] or the vaccine for SARS-CoV-19 [129]. With its effective network structure in terms of follower-, like-, and retweet-networks, Twitter provides an ideal environment for the empirical study of the spread of ideas.

The formation of echo-chambers has been well studied on Twitter and Facebook. Echo-chambers tend to reinforce like-mindedness in users, and tend as well to enable the crafting of a shared narrative [41]. The authors of [41] analysed the different ways in which different social media platforms’ algorithms influence the mechanisms of formation. They defined the echo-chambers based on the distributions of leanings towards polar attitudes. These attitude distributions were found to range from monomodal to bimodal or more complex. Regardless, polarisation is rarely neutral, and tends to favour opposition between extreme opinions. According to their results, Twitter and Facebook showed the most striking echo-chambers. Using virality models, they also measured information spread. In Twitter and Facebook, information was most likely to be spread to other users sharing similar leanings. Similar findings were shown by [130] by following the online debates surrounding vaccination hesitancy in Italy. Despite the formation of distinct echo-chambers, they found that community structure within echo-chambers also differed between vaccine advocates and sceptics and influenced information flow. Findings like these and others on polarised social network dynamics inspired us to analogise the model explored in the current work to online digital social media like Twitter, as well as to study how network structures influence echo-chamber formation. Alongside this, we chose to embrace an underlying active inference model as a cognitively inspired, Bayesian model for single agents’ belief formation.

To formalise confirmation bias as a fundamentally Bayesian phenomenon, we constructed our generative model to include a precision parameter that we named *epistemic conformation bias* or ECB. Specifically, ECB confers a higher weight to information that comes from peers that the reader (focal agent) believes are like-minded. This in turns leads an agent with higher ECB to selectively sample information that justifies what they already believe. We were able to replicate the formation of epistemic communities in silico, e.g., echo-chambers, on social networks such as Twitter. This unique formulation of confirmation bias as an epistemic phenomenon helps explain how individuals continuously forage their environment for information, but may become stuck in a so-called ‘bad bootstrap’ that simply reinforces existing beliefs about the world, which in the face of new information may lead to sub-optimal behaviour [131].

In agreement with previous work studying the relationship between synchronisation and network structure, we found that opinion dynamics depend heavily on network density. Our formalism allowed us to systematically vary the parameters of individual agents (e.g., cognitive biases or beliefs) as well as collective properties such as network structure. We found that the density of inter-agent connections, parameterised by connection probability of random graphs, determined the transition between echo-chamber formation (polarisation) and consensus. However, we found that in the presence of high ECB, one could observe polarisation even in the presence of dense connectivity (cf. Figure 4). This result seems counterintuitive, as we might think that network clustering is a necessary condition for more polarisation. However, clearly defined clusters and group boundaries can sometimes act as buffers [132,133,134,135,136]. Sub-clusters exchanging information are likely to average towards their local centre [137,138,139], which entails a form of opinion stability within the group. They are generally sheltered from other opinions since they cut ties to other agents not part of their group, and have been selected out [140]. However, in networks without clusters, opinions can have a high degree of volatility and reach very polar tendencies even without being entirely clustered. By means of epistemic confirmation bias, agents were likely to give more weight to information that was similar to their own, even in the presence of network neighbours with different opinions.

The clustering phenomenon is exacerbated by adding the capacity to form habits. Specifically, we allowed agents to increase their likelihood of resampling the same agents based on how often they attended to them in the past. Since neighbour-attendance is driven by epistemic value (resolving uncertainty about the $s^{\mathrm{Idea}}$ and $s^{\mathrm{MB}}$ hidden state factors), this tendency to revisit previously sampled neighbours is a form of ‘epistemic habit formation,’ where actions that are initially undertaken based on information gain become solidified over time due to a Pavlovian, model-free mechanism that simply reinforces past behaviour. We found that in addition to ECB, the presence of habit formation exacerbated polarisation, presumably due to the formation of echo-chambers or tight communities of agents that read only the Hashtag content of their like-minded peers. On the other hand, we found that beliefs about social volatility (represented by our $\omega^{\mathrm{Soc}}$ parameter) pushed the agents to sample their social environments more frequently and diversely, counteracting the effect of confirmation bias and habit formation in driving polarisation. We speculate that increased social volatility increases each agent’s incentive to sample a diverse array of network neighbours, which in turns lessens their susceptibility to believing in one **Idea** with high certainty. In other words, increased social volatility (low $\omega^{\mathrm{Soc}}$) makes agents more ‘curious’ about the beliefs of (potentially non-like-minded) neighbours, which in turns increases their exposure to conflicting information and ‘protects’ them from falling into one or another echo-chamber.

The contributing influence of beliefs about social volatility to exploratory social sampling leads us to consider the role of norms in social settings. If an agent is incentivised (via, e.g., epistemic value or curiosity) to pay attention to neighbours about whom they are uncertain, their social group could be a source of constant surprise, as long as their beliefs about their neighbours are constantly fickle (“I’m not sure what members of my social group believe from one time to the next”). In other words, even in the presence of a group of like-minded peers, we would expect that increased beliefs about social volatility leads to repeated attendance of peers among one another, even if those peers all agree (and believe as much about each other).

## 6. Conclusions

Our simulation showcased a novel opinion dynamics model based on multi-agent active inference, and highlights many interesting possibilities for future research. We introduced a new parameter, the epistemic confirmation bias, which can modulate the formation of epistemic communities by changing epistemic value in a biased way, namely towards attending preferentially to like-minded agents. In addition to the ECB, we also showed the importance of other features such as network structure and habit formation in contributing to polarised dynamics. However, there are several limitations to this work which warrant further discussion. While we systematised our study design to explore several parameters simultaneously, this search was not exhaustive and vast regions of parameter space remain unexplored. Particular parameters such as the size of the network and the ‘inverse environmental volatility’ $\omega^{\mathrm{Idea}}$ remained unexplored (we mainly explored networks with size N=30 and always fixed $\omega^{\mathrm{Idea}}=9.0$), and for computational efficiency we restricted both the resolution and the combinatorics of the parameter combinations explored. Network size is a major computational bottleneck, and thus our results are not guaranteed to generalise to larger networks. In future work, we could leverage distributed computing or GPU-accelerated operations to explore both larger network sizes and parameter combinations. However, in model spaces with high enough dimensions, computational acceleration alone will not suffice; one could thus also reduce the sampled region of parameter space by leveraging efficient search techniques (e.g., optimal experimental design [141]) or higher-order learning methods such as Bayesian hyperparameter optimisation [142]. The ‘inverse environmental volatility’ parameter $\omega^{\mathrm{Idea}}$ deserves further mention: as explained in Section 2.5, $\omega^{\mathrm{Idea}}$ encodes the precision of the transition model $B^{\mathrm{Idea}}$. This can be understood as the the agent’s beliefs about the uncertainty characterising the truth status of the ‘Idea‘ itself. We intentionally fixed this parameter to a constant value of $\omega^{\mathrm{Idea}}=9.0$ for all simulations. In other words, all agents believed that the $s^{\mathrm{Idea}}$ hidden state changed very slowly or with low uncertainty. As mentioned above, we made this simplification for the current study to limit the volume of the parameter space explored. Additionally, exploring beliefs about the dynamics of $s^{\mathrm{Idea}}$ was not particularly relevant to the current study, because we did not include any true hidden environmental dynamics in the simulations. In fact, the agents only observe the behaviour of other agents, and never observe any true signal from the environment itself. Including a veridical, non-social signal from the environment, however, would be an interesting extension for future work.

The generative model used by the single agents was also limited, in the sense that we only modelled beliefs in one of two mutually exclusive Ideas. Previous research into opinion or collective dynamics has shown that such binarity may strongly determine the dynamics of the system [104,143]. From a construct-validity standpoint, such binarity also vastly simplifies the semantic complexity found in real epistemic communities. For example, the semantic expression of a particular idea or claim heavily depends on the community in which it circulates. In future designs, we should strive to make the ideas more complex and more porous. By porosity we mean ‘semantic cross-over’, in the sense that multiple ideas may entail more or less similar behavioural consequences, or indeed entail the truth value of one another. This porosity may give rise to groups that believe in the same idea from an inference standpoint, but have a different interpretation of it. Starting from there, we can begin to envisage a specific semantic embedding which leads us to social scripts [144]. These conceptual embeddings would lead to two different conceptions with distinct causal relations to the environment. The weak conception of the script corresponds to an embedding, linking the observation of an event to the belief in a particular idea. The strong conception of the script leads to a sequencing of the beliefs, such as an entailment relation (e.g., ‘if I believe X, this entails a belief in Y’). This type of conceptual entailment possible under a strong conception of social scripts, combined with the ability to express one’s beliefs, could engender a capacity to act and coordinate through language with other actors.

Future work could explicitly model these entailment relationships among semantic entities by violating the typical independence assumption used to factorise the generative model’s hidden state factors—for instance, instead of having each hidden state factor $s^{\left(f\right)}$ being conditionally dependent on only other states/control states *within that factor*, we could ‘mix’ hidden state factors to make states of factor *i* depend on states of factor *j*.

Another notable feature to include is the variation of prior beliefs about different ideas or claims. In the current model, agents were often initialised to have uniformly distributed beliefs about $s^{\mathrm{Idea}}$ around the ‘ambivalence’ line of [0.5,0.5]. Future studies could quantitatively investigate the dependence of epistemic community formation on the initial distribution of prior beliefs and how that distribution intersects with structural features such as network position (e.g., ‘is a very confident agent more influential in determining information spread, when it’s a peripheral vs. central node in the network?’). In this way, we could study ‘historical effects’ such as whether pre-existing echo-chambers or belief distributions influence the susceptibility of the network to incoming information or environmental fluctuations.

Another limitation of this work is the relative simplicity of the dependent measurements we used to characterise collective outcomes. For instance, we measured the degree of collective consensus vs. polarisation through the polarisation metric. This metric is a scalar which quantifies the degree to which the entire network believes in one Idea vs. is split into believing in two Ideas. However, this one-dimensional metric is ambiguous with respect to exactly *how* beliefs are spread throughout the network, in the case of low consensus—for instance, a low polarisation metric does not disambiguate whether all the agents that believe in one Idea have network connections to each other, or whether they are isolated and only ‘by chance’ believe in the same Idea. Future investigations should thus develop more sensitive metrics, depending on the hypothesis, that take into consideration how individual beliefs correlate with the network topology. In addition, we could calculate polarisation-style metrics using other dimensions of the agents’ beliefs—for example, we could measure polarisation using the so-called ‘meta-beliefs,’ in addition to just the agents’ beliefs about the Idea itself.

In future studies, we hope to investigate individual cognitive differences more quantitatively using the active inference framework. Under active inference, ‘individual differences’ can be formalised as variance among the parameters of generative models across agents—e.g., different settings of the inverse volatility parameters for different agents. Another interesting possibility that is accommodated within the active inference framework is the idea that agents may *learn* the parameters of their generative models, as opposed to keeping them fixed over time. For example, one could imagine that the epistemic confirmation bias associated with a particular neighbour *k* could change over time as a function of the reliability of Hashtags observed by the focal agent. This is easily cast as another form of inference under the Bayesian framework. All one would need to do is define appropriate priors and approximate posteriors over γ, from which an additional free energy term and appropriate belief updating scheme could be derived. Learning the parameters may add ecological validity to the model as well; for example, agents might become accustomed to their social environment and seek out an epistemic community in order to increase the predictability of their sensory information, thus requiring them to sample their social environment less frequently. This is the kind of phenomenon that could be modelled by letting the inverse social volatilities $\omega^{\mathrm{Soc}}$ become free, learnable parameters. With larger networks, we may be able to simulate the emergence of similar but distant sub-communities, which become epistemically similar without coming into direct contact, or only through very distant contact with one another. This leads us to the possibility of simulating the way epistemic and pragmatic practices become cemented, giving way to social meaning semantics and scripts, which seem to separate cultures. Simulating the emergence of similar semantics and scripts across different communities may help us further understand their common underlying processes. Finally, in future studies, we could model an explicit state of conformity, by modelling the agent’s assumptions about the groups they can identify around themselves, and be driven to model their behaviour after the group they feel most kinship to.

## Figures and Tables

**Table 1:** Variables of the POMDP generative model of single-agent opinion formation. The abstract name of each variable is written in the left column, its mathematical notation is in the middle column, and the right column shows how these variables correspond to different components of the opinion formation generative model. *M* is the total number of observation modalities and *F* is the number of hidden state/control factors. The observation model is a categorical likelihood distribution encoded by A, which comprises a collection of modality-specific $A^{\left(m\right)}$ arrays. The transition model is also a likelihood, mapping each state to its successor in time, encoded by the $B^{\left(f\right)}$ arrays. The initial distribution over hidden states is encoded by the D vector, and the prior distribution over control factors is encoded by the E and ε distributions.

Variable Name	Notation	Meaning
		The focal agent’s tweets $o^{\mathrm{ST}}\in Z^{1\times H}$
**Observations**	$o=\{o^{\left(1\right)},\ldots ,o^{\left(M\right)}\}$	Neighbour *k*’s tweets $o^{\mathrm{NT}k}\in Z^{1\times (H+1)}$
		The sampled agent $o^{\mathrm{Who}}\in Z^{1\times K}$
		The focal agent’s beliefs $s^{\mathrm{Idea}}\in Z^{1\times 2}$
**Hidden States**	$s=\{s^{\left(1\right)},\ldots ,s^{\left(F\right)}\}$	Neighbour *k*’s beliefs $s^{\mathrm{MB}k}\in Z^{1\times 2}$
		The Hashtag tweeted by focal agent $s^{T}\in Z^{1\times H}$
		The neighbour sampled focal agent $s^{\mathrm{Who}}\in Z^{1\times n}$
**Actions**	$u=\{u^{\left(1\right)},\ldots ,u^{\left(F\right)}\}$	The Hashtag control state $u^{T}\in Z^{1\times H}$
		The neighbour attendance control state $u^{\mathrm{Who}}\in Z^{1\times n}$
		Self tweet likelihood $A^{\mathrm{ST}}\in {\left(R_{>0}\right)}^{2\times 2\times 2K\times H\times K}$
**Observation model**	$P\left(o_{t}^{\left(m\right)}=i|s_{t}^{\left(1\right)}=j,s_{t}^{\left(2\right)}=k,\ldots \right)={\left[A^{\left(m\right)}\right]}_{ijk\ldots }$	Neighbour tweet likelihood $A^{\mathrm{NT}k}\in {\left(R_{>0}\right)}^{2\times 2\times 2K\times H\times K}$
		Neighbour attend likelihood $A^{\mathrm{Who}}\in {\left(R_{>0}\right)}^{K\times 2\times 2K\times H\times K}$
		Environmental dynamics and volatility $B^{\mathrm{Idea}}\in R_{>0}^{2\times 2}$
**Transition model**	$P\left(s_{t+1}^{\left(f\right)}=i|s_{t}^{\left(f\right)}=j,u_{t}^{\left(f\right)}=k\right)={\left[B^{\left(f\right)}\right]}_{ijk}$	Meta-belief dynamics and volatility $B^{\mathrm{MB}k}\in {\left(R_{>0}\right)}^{2\times 2}$
		Tweet control $B^{T}\in {\left(R_{>0}\right)}^{H\times H\times H}$
		Neighbour attendance control $B^{\mathrm{Who}}\in {\left(R_{>0}\right)}^{K\times K\times K}$
**Initial State**	$p\left(s_{0}^{\left(f\right)}=i\right)={\left[D^{\left(f\right)}\right]}_{i}$	Initial state distribution $D\in {\left(R_{>0}\right)}^{2\times 2K\times H\times K}$
**Control State Prior**	$P\left(u_{0}^{T}|s^{\mathrm{idea}}\right)=E^{T}$	Empirical prior over Hashtag control state $E^{T}\in {\left(R_{>0}\right)}^{H\times 2}$
	$P\left(u_{0}^{\mathrm{Who}}|E^{\mathrm{Who}}\right)=E\left[Dir\left(\varepsilon \right)\right]$	Dirichlet hyperparameters over neighbour attendance control state $\varepsilon \in {\left(R_{>0}\right)}^{1\times K}$

## Appendix A

In this appendix we provide additional mathematical details on the update equations for perception and action (policy inference) under active inference.

We begin by recalling the POMDP generative model and the approximate posterior over hidden states and policies:

(A1) $$\begin{array}{cc} P\left(\tilde{o},\tilde{s},\tilde{u},\pi \right) & =P\left(s_{1}\right)P\left(\pi \right)\prod_{\tau =2}^{T}P\left(s_{\tau }|s_{\tau -1},u_{\tau }\right)P\left(u_{\tau }|\pi \right)\prod_{\tau =1}^{T}P\left(o_{\tau }|s_{\tau }\right)\\ Q(s_{\tau }|\pi ) & =Cat(s_{\pi \tau })\\ Q(\pi ) & =Cat(\pi )\\ Q(s_{1:T},\pi ) & =Q\left(\pi \right)\overset{T}{\prod_{\tau =1}}Q\left(s_{\tau }|\pi \right) \end{array}$$

Given this generative model and approximate posterior, we can now write down the variational free energy over time:

$$\begin{array}{cc} F_{1:T} & =E_{Q(s_{1:T},\pi )}\left[\ln Q\left(s_{1:T},\pi \right)-\ln P\left(o_{1:T},s_{1:T},\pi \right)\right] \end{array}$$

Due to the Markovian nature of the POMDP generative model and the factorised form of the posterior, the free energy over trajectories can be factorised into a per-timestep free energy $F_{\tau }$, which has the following simple form:

(A2) $$\begin{array}{cc} F_{\tau } & =E_{Q\left(s_{\tau }|\pi \right)Q\left(\pi \right)}\left[\ln Q\left(s_{\tau }|\pi \right)-\ln P\left(o_{\tau },s_{\tau }|s_{\tau -1},\pi \right)\right] \end{array}$$

In the following subsections we show how state estimation (perception) and policy inference (decision-making/planning) are derived by minimising the variational free energy functionals with respect to the parameters of the posterior $Q(s_{1:T},\pi )$.

### Appendix A.1. State Estimation

State state estimation consists of optimising the $Q(s_{1:T}|\pi )$ over hidden states under different policies. Because our approximate posterior and generative model are defined using categorical distributions, the problem of state estimation becomes minimising free energy gradients of the form $\frac{\partial F}{\partial s}$, where s are the parameters of the approximate posterior distribution over hidden states, Q(s)=Cat(s), where the notation P(x)=Cat(ϕ) denotes a categorical distribution over some random variable *x* with parameters ϕ.

In the ‘full construct’ version of active inference (see, e.g., [86]), the full joint posterior $Q(s_{1:T},\pi )$ is optimised simultaneously, meaning that the posterior over hidden states is conditioned on policies. This means that the full posterior beliefs at any timestep *t* include a separate $Q(s_{t}|\pi )$ under each policy, where the beliefs about a given timestep under a given policy are often denoted by the sufficient statistics $s_{\pi \tau }$. For the current model, we have simplified posterior inference to rely on an approximate posterior where hidden states are independent of policies. This move is justified because the practical differences between the ‘full construct’ and simplified versions are negligible, in the limit of small policy spaces and short time horizons (such as in the current work). Therefore in the current study we amended the variational posterior to have the following form:

$$\begin{array}{c} Q\left(s_{1:T},\pi \right)=Q\left(\pi \right)\prod_{\tau =1}^{T}Q\left(s_{\tau }\right) \end{array}$$

Given this simplified posterior, state estimation proceeds by optimising the posterior belief about hidden states at the current timestep $Q^{*}\left(s_{t}\right)$ using the current observation $o_{t}$. This can be found using the gradients of the variational free energy from Equation (A2), now using the simplified form of the posterior:

(A3) $$\begin{array}{cc} F_{t} & =E_{Q\left(s_{t}\right)Q\left(\pi \right)}\left[\ln Q\left(s_{t}\right)-\ln P\left(o_{t},s_{t}|s_{t-1},\pi \right)\right]\\ \Rightarrow \frac{\partial F_{t}}{\partial Q(s_{t})} & =0\Leftrightarrow Q^{*}\left(s_{t}\right)=\sigma \left(\ln P\left(o_{t}|s_{t}\right)+\ln\left(E_{P(s_{t-1},u_{t-1})}\left[P\left(s_{t}|s_{t-1},u_{t-1}\right)P\left(s_{t-1}\right)\right]\right)\right) \end{array}$$

where $\sigma \left(x\right)=\frac{e^{x}}{\sum_{x}e^{x}}$ is the normalised exponential or softmax function. Equation (A3) is a type of ‘fixed-point solution’ for the posterior, where the beliefs about hidden states are directly set to the solution of minimal free energy (where $\frac{\partial F_{t}}{\partial Q(s_{t})}=0$). Note that this differs with the classic ‘gradient descent’ scheme used to optimise the variational posterior with marginal message passing or variational message passing, as proposed in [86], which was originally invoked as a biologically plausible update scheme that could be implemented by neuronal population dynamics. Since we are not interested in simulating neurophysiological responses and the belief updating is simpler, for the simulations presented in this paper, we used this simpler update rule.

The functional form of (A3) invites a straightforward Bayesian interpretation: the ‘best’ posterior belief $Q^{*}\left(s_{t}\right)$ is proportional to the product of a likelihood term $P(o_{t}|s_{t})$ and a prior term $P\left(s_{t}|s_{t-1},u_{t-1}\right)P\left(s_{t-1}\right)$—the definition of Bayes rule. In practice, we use a ‘moving empirical prior’ rule, where the posterior from last timestep’s optimisation $Q^{*}\left(s_{t-1}\right)$ becomes the prior $P(s_{t-1})$. This means the update rule can be re-written as follows:

(A4) $$\begin{array}{cc} P(s_{t-1}) & \approx Q(s_{t-1})\\ \Rightarrow Q^{*}\left(s_{t}\right) & =\sigma (\ln P\left(o_{t}|s_{t}\right)+\ln E_{Q(s_{t-1})}\left[P\left(s_{t}|s_{t-1},u_{t-1}\right)\right]) \end{array}$$

This means that at each timestep, the current posterior is a Bayesian average between the likelihood term and the previous timestep’s posterior belief, passed through the action-conditioned transition dynamics $P(s_{t}|s_{t-1},u_{t-1})$ of the generative model. Note that this update rule can be extended to generative models with factorised observations o and hidden states s by rewriting Equation (A4) for a particular marginal $Q^{*}\left(s_{t}^{f}\right)$ as follows:

(A5) $$\begin{array}{c} Q^{*}\left(s_{t}^{f}\right)=\sigma \left(E_{Q^{i\setminus f}}\left[\ln P(o_{t}|s_{t})\right]+\ln E_{Q(s_{t-1}^{f})}\left[P\left(s_{t}^{f}|s_{t-1}^{f},u_{t-1}\right)\right]\right) \end{array}$$

where the expectation $E_{Q^{i\setminus f}}$ denotes an expectation with respect to all posterior marginals $Q(s_{t}^{i})$ besides the marginal $Q(s_{t}^{f})$ currently being optimised. To find the full, multi-factor posterior $Q^{*}\left(s_{t}\right)$, this equation is iterated across marginals, holding the existing solutions for all other marginals fixed while a particular one is updated [126].

### Appendix A.2. Policy Inference

Under active inference, policies π inferred, i.e., the agent optimises a variational posterior over policies denoted by Q(π). The optimal posterior over policies $Q^{*}\left(\pi \right)$ is obtained by minimising the free energy with respect to the categorical parameters π (cf. Equation (A1)). This can be shown by rewriting the full variational free energy over time $F_{1:T}$ as the sum of a complexity term that measures the divergence between the posterior and prior over policies, and an expected ‘accuracy’-like term:

(A6) $$\begin{array}{cc} F_{1:T} & =E_{Q(s_{1:T},\pi })\left[\ln Q\left(s_{1:T},\pi \right)-\ln P\left(o_{1:T},s_{1:T},\pi \right)\right]\\ \; & =E_{Q(s_{1:T},\pi )}\left[\ln Q\left(\pi \right)+\sum_{\tau =1}^{T}\ln Q\left(s_{\tau }|\pi \right)-\ln P\left(\pi \right)-\ln P\left(o_{1:T},s_{1:T}|\pi \right)\right]\\ \; & =D_{KL}\left[Q\left(\pi \right)\|P\left(\pi \right)\right]+E_{Q(\pi )}\left[F(\pi )\right] \end{array}$$

where the second term is the expected variational free energy of policies F(π), which is defined as follows:

(A7) $$\begin{array}{cc} F(\pi ) & =-E_{Q(s_{1:T}|\pi )}[\ln P\left(o_{1:T},s_{1:T}|\pi \right)-H\left[Q\left(s_{1:T}|\pi \right)\right] \end{array}$$

The optimal posterior that minimises the full variational free energy F is found by taking the derivative of F with respect to Q(π) and setting this gradient to 0, yielding the following free-energy-minimising solution for Q(π):

(A8) $$\begin{array}{cc} Q^{*}\left(\pi \right) & =\underset{\pi }{\mathrm{argmin}}\;F=\sigma \left(\ln P\left(\pi \right)-F\left(\pi \right)\right) \end{array}$$

Therefore in the same way that state estimation or optimisation of Q(s) in Equation (A4) resembles a Bayesian average of a likelihood and a prior term, policy inference also becomes an average of the policy prior P(π) and the ‘evidence’ afforded to each policy, scored by F(π). Recall here that the policy prior P(π) is itself decomposed as a combination of the expected free energy prior and the ‘habit vector’: $P\left(\pi \right)=P\left(\pi_{0}\right)-G$.

## Appendix B

In this appendix derive a quantitative relationship between the epistemic confirmation bias γ and the negative ambiguity term of the epistemic value. Recall the definition of the epistemic value:

$$\begin{array}{cc} E_{Q(o_{\tau }|\pi )}\left[D_{KL}\left(Q\left(s_{\tau }|o_{\tau },\pi \right)\right\|Q\left(s_{\tau }|\pi \right)\right] & =-E_{Q(s_{\tau }|\pi )}\left[H[P\left(o_{\tau }|s_{\tau }\right)]\right]+H\left[Q\left(o_{\tau }|\pi \right)\right] \end{array}$$

We define the first term on the RHS of the decomposition as the negative ambiguity $H=-E_{Q(s_{\tau }|\pi )}\left[H[P\left(o_{\tau }|s_{\tau }\right)]\right]$. We drop the τ subscript hereafter for simplicity, and restrict ourselves only to the computation of this term for the neighbour *k* tweet observation modality $o^{\mathrm{NT}k}$ and the hidden states that it depends on, $s^{\mathrm{Idea}}$ and $s^{\mathrm{MB}k}$. We further condition our analysis only on those policies that entail sampling neighbour *k*, i.e., those policies where $s^{\mathrm{Who}}=u^{\mathrm{Who}}=k$. Therefore we redefine Q(s) here as $Q(s^{\mathrm{Idea}},s^{\mathrm{MB}k})$.

**Theorem** **A1.**
*The negative ambiguity is proportional to the epistemic confirmation bias parameter γ.*

$$H\propto k^{\gamma }\log k^{\gamma }$$

**Proof.** WLOG, we simplify the state space to only consider the states $s^{\mathrm{Idea}}$ and $s^{\mathrm{MB}k}$ and the observation of neighbour *k*’s Hashtag $o^{\mathrm{NT}k}$. Recall that observation likelihood for $o^{\mathrm{NT}k}$, in the case that $s^{\mathrm{Idea}}=s^{\mathrm{MB}k}$ is as a softmax transformation of (certain columns of) the base likelihood $P(o^{\mathrm{NT}k}|s^{\mathrm{Idea}},s^{\mathrm{MB}k})$ and the epistemic confirmation bias parameter γ:

(A9) $$P\left(o^{\mathrm{NT}k}|s^{\mathrm{Idea}}=s^{\mathrm{MB}k},\gamma \right)=\frac{e^{\gamma P(o^{\mathrm{NT}k}|s^{\mathrm{Idea}}=s^{\mathrm{MB}k}))}}{\sum e^{\gamma P(o^{\mathrm{NT}k}|s^{\mathrm{Idea}}=s^{\mathrm{MB}k}))}}$$

In this case, the likelihood $P(o^{\mathrm{NT}k}|s^{\mathrm{MB}k})$ is comprised of two Bernoulli distributions, where

$$P\left(o^{\mathrm{NT}k}|s^{\mathrm{MB}k}\right)=\left\{\mathrm{Bern}\left(p_{h}\right),\mathrm{Bern}\left(1-p_{h}\right)\right\}$$

where $p_{h}$ is the ‘Hashtag reliability’ parameter of the matrix h:

$$h=\left[\begin{array}{cc} p_{h} & 1-p_{h}\\ 1-p_{h} & p_{h} \end{array}\right]$$

where this matrix is ‘copied‘ across the dimension of the likelihood corresponding to the two settings of $s^{\mathrm{Idea}}$. Note also that the posterior over hidden states is factorised into two independent marginal posteriors.

(A10) $$Q\left(s^{\mathrm{Idea}},s^{\mathrm{MB}k}\right)=Q\left(s^{\mathrm{Idea}}\right)Q\left(s^{\mathrm{MB}k}\right)$$

The definition of negative ambiguity is

(A11) $$\begin{array}{c} H=-E_{Q(s|\pi )}\left[H\left[P\left(o^{\mathrm{NT}k}|s\right)\right]\right] \end{array}$$

Furthermore, we can write the negative entropy of the given likelihood as

$$-H\left[P\left(o^{\mathrm{NT}k}|s^{\mathrm{MB}k}\right)\right]=p_{h}\log p_{h}+\left(1-p_{h}\right)\log\left(1-p_{h}\right)$$

Using Equation (A9) we have

$$-H\left[P\left(o^{\mathrm{NT}k}|s^{\mathrm{MB}k}=s^{\mathrm{Idea}}\right)\right]=\frac{p_{h}^{\gamma }}{C}\log\frac{p_{h}^{\gamma }}{C}+\frac{{\left(1-p_{h}\right)}^{\gamma }}{C}\log\frac{{\left(1-p_{h}\right)}^{\gamma }}{C}$$

where $C=p^{\gamma }+{\left(1-p\right)}^{\gamma }$ The negative entropy can be decomposed into a sum of negative entropies:

$$-H\left[P\left(o^{\mathrm{NT}k}|s^{\mathrm{MB}k}\right)\right]=-H\left[P\left(o^{\mathrm{NT}k}|s^{\mathrm{MB}k}=s^{\mathrm{Idea}}\right)\right]-H\left[P\left(o^{\mathrm{NT}k}|s^{\mathrm{MB}k}\neq s^{\mathrm{Idea}}\right)\right]$$

which then means the total negative ambiguity can be written as follows, expanding the expressions for the entropies in terms of the Bernoulli parameter $p_{h}$:

(A12) $$\begin{array}{c} H=E_{Q(s^{\mathrm{Idea}},s^{\mathrm{MB}k})}[\frac{1}{C}\left[p_{h}^{\gamma }\log p_{h}^{\gamma }+{\left(1-p_{h}\right)}^{\gamma }\log{\left(1-p_{h}\right)}^{\gamma }-2\log C\right]\\ +[p_{h}\log p_{h}+\left(1-p_{h}\right)\log\left(1-p_{h}\right)]] \end{array}$$

Since all terms in Equation (A12) are increasing in γ insofar as $p_{h}\geq 0$, then negative ambiguity is directly proportional to γ. □

**Theorem** **A2.**
*For any γ>1, and for any realisation of the posterior $Q(s^{\mathrm{Idea}},s^{\mathrm{MB}k})$ the negative ambiguity will be maximised in the case that the posterior beliefs about $s^{\mathrm{Idea}}$ and the posterior beliefs about $s^{\mathrm{MB}k}$ are either both greater than 0.5 or both less than 0.5.*

∀γ>1

$$\begin{array}{c} \max H\in \{H:\mathrm{sgn}\left(Q\left(\left(s^{\mathrm{Idea}}\right)-0.5\right)=\mathrm{sgn}\left(Q\left(s^{\mathrm{MB}k}\right)-0.5\right)\right\} \end{array}$$

**Proof.** We now use the fact that $Q(s^{\mathrm{Idea}})$ and $Q(s^{\mathrm{MB}k})$ are also Bernoulli probability distributions, such that

$$\begin{array}{c} Q\left(s^{\mathrm{Idea}}\right)=\mathrm{Bern}\left(p\right)\\ Q\left(s^{\mathrm{MB}k}\right)=\mathrm{Bern}\left(q\right) \end{array}$$

We also note that since the γ parameter only scaled the likelihood in the case that $s^{\mathrm{Idea}}=s^{\mathrm{MB}k}$, the values of the posterior that these correspond to are only those along the diagonal of the joint probability distribution of $Q(s^{\mathrm{Idea}},s^{\mathrm{MB}k})$, namely the joint line of solutions through *p* and *q* connected by the points qp and (1−q)(1−p). Expanding the expectation in Equation (A12), we can write the negative ambiguity as

(A13) $$\begin{array}{c} -qp\left(H\left[P\left(o^{\mathrm{NT}k}|s^{\mathrm{MB}k}=s^{\mathrm{Idea}}\right)\right]\right)-\left(1-q\right)\left(1-p\right)\left(H\left[P\left(o^{\mathrm{NT}k}|s^{\mathrm{MB}k}=s^{\mathrm{Idea}}\right)\right]\right)\\ -q\left(1-p\right)\left(H\left[P\left(o^{\mathrm{NT}k}|s^{\mathrm{MB}k}\neq s^{\mathrm{Idea}}\right)\right]\right)-p\left(1-q\right)\left(H\left[P\left(o^{\mathrm{NT}k}|s^{\mathrm{MB}k}\neq s^{\mathrm{Idea}}\right)\right]\right) \end{array}$$

Suppose that γ>1. This means that for nonzero $p_{h}$, the entropy terms in Equation (A13) will be exponentiated by a power greater than 1, which implies that

(A14) $$H\left[P\left(o^{\mathrm{NT}k}|s^{\mathrm{MB}k}=s^{\mathrm{Idea}}\right)\right])<H\left[P\left(o^{\mathrm{NT}k}|s^{\mathrm{MB}k}\neq s^{\mathrm{Idea}}\right)\right])$$

Now take the case that $Q(s^{\mathrm{Idea}})>0.5$ and $Q(s^{\mathrm{MB}k})>0.5$. This means that the largest coefficient scaling the entropy of the likelihood will necessarily be qp, which scales $-H[P\left(o^{\mathrm{NT}k}|s^{\mathrm{MB}k}=s^{\mathrm{Idea}}\right)])$. Similarly, if $Q(s^{\mathrm{Idea}})<0.5$ and $Q(s^{\mathrm{MB}k})<0.5$, the largest coefficient scaling the entropy of the likelihood will necessarily be (1−p)(1−q), which also scales $-H[P\left(o^{\mathrm{NT}k}|s^{\mathrm{MB}k}=s^{\mathrm{Idea}}\right)])$. However, if $\mathrm{sgn}(Q\left(\left(s^{\mathrm{Idea}}\right)-0.5\right)\neq \mathrm{sgn}\left(Q\left(s^{\mathrm{MB}k}\right)-0.5\right)$, the largest coefficients will be either (1−p)q or p(1−q) which will be scaling $-H[P\left(o^{\mathrm{NT}k}|s^{\mathrm{MB}k}\neq s^{\mathrm{Idea}}\right)]$. Therefore, because of Equations (A13) and (A14), the maximum negative ambiguity for any value of γ>1 will always be reached when $\mathrm{sgn}(Q\left(\left(s^{\mathrm{Idea}}\right)-0.5\right)=\mathrm{sgn}\left(Q\left(s^{\mathrm{MB}k}\right)-0.5\right)$. □

Figure A1 provides a visual intuition for the relationship between the two marginal posteriors (defined by Bernoulli parameters *p* and *q*), the epistemic confirmation bias γ and the components of the epistemic value, decomposed here as the negative ambiguity $H=E_{Q(s|\pi )}\left[H\left[P\left(o|s\right)\right]\right]$ and the entropy of the predictive distribution over observations Q(o|π).

**Theorem** **A3.**
*The inverse social volatility $\omega^{\mathrm{Soc}}$ is inversely related to the epistemic value of policies that entail sampling a particular neighbour-in other words $EV\propto \frac{1}{\omega^{\mathrm{Soc}}}$.*

**Proof.** Recall the decomposition of the expected free energy in the Section 2.11 for a policy π into the negative instrumental value and the negative salience or epistemic value. For convenience, we define a pseudo-‘value’ function for policies as the negative of the expected free energy V(π)≡−G(π):

(A15) $$\begin{array}{cc} V(\pi ) & =\underset{\mathrm{Instrumental}\;\mathrm{value}}{\underset{︸}{E_{Q\left(o_{\tau }|\pi \right)}\left[\ln\tilde{P}\left(o_{\tau }\right)\right]}}+\underset{\mathrm{Epistemic}\;\mathrm{value}}{\underset{︸}{E_{Q\left(o_{\tau }|\pi \right)}\left[D_{KL}\left[Q\left(s_{\tau }|o_{\tau },\pi \right)∥Q\left(s_{\tau }|\pi \right)\right]\right]}} \end{array}$$

It is straightforward to show the positive relationship between the entropy of the policy-conditioned beliefs $H[Q\left(s_{\tau }|\pi \right)]$ and the epistemic value. We begin by isolating further analysis only to the meta-belief hidden state factor $s^{\mathrm{MB}k}$ for a particular neighbour *k* and the corresponding observation modality: $o^{\mathrm{NT}k}$. For notational convenience, we let $s=s^{\mathrm{MB}k}$ and $o=o^{\mathrm{NT}k}$. Using this notation, we can then rewrite the epistemic value as the predictive mutual information between states and observations, using the predictive distributions $Q(o_{\tau }|\pi )$ and $Q(s_{\tau }|\pi )$:

(A16) $$\begin{array}{cc} E_{Q\left(o_{\tau }|\pi \right)}\left[D_{KL}\left[Q\left(s_{\tau }|o_{\tau },\pi \right)∥Q\left(s_{\tau }|\pi \right)\right]\right] & =D_{KL}\left[Q\left(o_{\tau },s_{\tau }|\pi \right)∥Q\left(s_{\tau }|\pi \right)Q\left(o_{\tau }|\pi \right)\right]\equiv I_{pred}\left(O;S\right)\\ \; & =\sum_{o_{\tau },s_{\tau }}Q\left(o_{\tau },s_{\tau }|\pi \right)\ln\frac{Q\left(o_{\tau },s_{\tau }|\pi \right)}{Q\left(s_{\tau }|\pi \right)Q\left(o_{\tau }|\pi \right)}\\ \; & =-H\left[Q\left(o_{\tau },s_{\tau }|\pi \right)\right]+H_{Q(o_{\tau },s_{\tau }|\pi )}\left[Q\left(o_{\tau }|\pi \right)\right] \end{array}$$

(A17) $$\begin{array}{cc} \; & \;-\sum_{o_{\tau },s_{\tau }}Q\left(o_{\tau },s_{\tau }|\pi \right)\ln Q\left(s_{\tau }|\pi \right) \end{array}$$

Using the factorisation of the joint posterior predictive density $Q\left(o_{\tau },s_{\tau }|\pi \right)=P\left(o_{\tau }|s_{\tau }\right)$$Q(s_{\tau }|\pi )$, the final term on the RHS of Equation (A17) can be rewritten:

(A18) $$\begin{array}{cc} -\sum_{o_{\tau },s_{\tau }}Q\left(o_{\tau },s_{\tau }|\pi \right)\ln Q\left(s_{\tau }|\pi \right) & =-\sum_{o_{\tau },s_{\tau }}P\left(o_{\tau }|s_{\tau }\right)Q\left(s_{\tau }|\pi \right)\ln Q\left(s_{\tau }|\pi \right) \end{array}$$

(A19) $$\begin{array}{cc} \; & \geq H\left[Q\left(s_{\tau }|\pi \right)\right]+E_{Q(s_{\tau }|\pi )}\left[\ln P\left(o_{\tau }|s_{\tau }\right)\right] \end{array}$$

where the inequality going from (A18) to (A19) follows from Jensen’s inequality. Equation (A19) demonstrates that uncertainty about hidden states (as quantified by $H[Q\left(s_{\tau }|\pi \right)]$) is directly proportional to the drive to reduce that uncertainty, subject to the log probability of observations expected under hidden states $E_{Q(s_{\tau }|\pi )}\left[\ln P\left(o_{\tau }|s_{\tau }\right)\right]$. We can then use the dependence of $P(s_{\tau }|s_{\tau -1},\pi )$ on $\omega^{\mathrm{Soc}}$ to relate the inverse social volatility to the posterior entropy $H[Q\left(s_{\tau }|\pi \right)]$.

(A20) $$\begin{array}{cc} Q(s_{\tau }|\pi ) & =P\left(s_{\tau }|s_{\tau -1},\pi ,\omega^{\mathrm{Soc}}\right)Q\left(s_{\tau -1}|\pi \right)=\frac{e^{\omega^{\mathrm{Soc}}P\left(s_{\tau }|s_{\tau -1},\pi \right)}}{\sum_{s_{\tau }}e^{\omega^{\mathrm{Soc}}P\left(s_{\tau }|s_{\tau -1},\pi \right)}}Q\left(s_{\tau -1}|\pi \right) \end{array}$$

(A21) $$\begin{array}{cc} \ln Q(s_{\tau }|\pi ) & =\omega^{\mathrm{Soc}}P\left(s_{\tau }|s_{\tau -1},\pi \right)-\ln\sum_{s}e^{\omega^{\mathrm{Soc}}P\left(s_{\tau }|s_{\tau -1},\pi \right)}+\ln Q\left(s_{\tau -1}|\pi \right) \end{array}$$

(A22) $$\begin{array}{cc} \ln Q(s_{\tau }|\pi ) & =\omega^{\mathrm{Soc}}P\left(s_{\tau }|s_{\tau -1},\pi \right)-C+\ln Q\left(s_{\tau -1}\right) \end{array}$$

(A23) $$\begin{array}{cc} E_{Q(s_{\tau }|\pi )}\left[\ln Q\left(s_{\tau }|\pi \right)\right] & =\omega^{\mathrm{Soc}}E_{Q(s_{\tau }|\pi )}\left[P\left(s_{\tau }|s_{\tau -1},\pi \right)\right]+E_{Q(s_{\tau }|\pi )}\left[\ln Q\left(s_{\tau -1}\right)-C\right] \end{array}$$

(A24) $$\begin{array}{cc} \Rightarrow H[Q\left(s_{\tau }|\pi \right)] & \propto -\omega^{\mathrm{Soc}}E_{Q(s_{\tau }|\pi )}\left[P\left(s_{\tau }|s_{\tau -1},\pi \right)\right] \end{array}$$

The final line demonstrates that the entropy of the predictive posterior is inversely proportional to the inverse social volatility $\omega^{\mathrm{Soc}}$, and thus controls the rate at which the focal agent’s uncertainty about their neighbours‘ belief-states increases, and therefore also determines the epistemic value of policies that entail reading that neighbour’s tweets. Intuitively, if an agent believes their social world is volatile (the beliefs of neighbouring agents quickly grow uncertain), the agent will become incentivised to sample those neighbours more frequently, in order to resolve rapidly growing uncertainty about $s^{\mathrm{MB}}$. Importantly, this epistemic value will grow over time for a particular neighbour as long as that neighbour’s tweets are not read, so a particular value of $\omega^{\mathrm{Soc}}$ entails a characteristic ‘social re-attendance rate’ for each neighbour. □
